# Impact of Annual Albendazole versus Four-Monthly Test-and-Treat Approach of Intestinal Parasites on Children Growth—A Longitudinal Four-Arm Randomized Parallel Trial during Two Years of a Community Follow-Up in Bengo, Angola

**DOI:** 10.3390/pathogens10030309

**Published:** 2021-03-07

**Authors:** Carolina Gasparinho, Aguinaldo Kanjungo, Félix Zage, Isabel Clemente, Ana Santos-Reis, Miguel Brito, José Carlos Sousa-Figueiredo, Filomeno Fortes, Luzia Gonçalves

**Affiliations:** 1 Centro de Investigação em Saúde de Angola (CISA), Rua Direita de Caxito, Caxito, Angola; aguinaldokanjungo@hotmail.com (A.K.); felix.zage@cisacaxito.org (F.Z.); isabel.clemente@outlook.com (I.C.); miguel.brito@estesl.ipl.pt (M.B.); josecarlos.figueiredo@gmail.com (J.C.S.-F.); 2 Global Health and Tropical Medicine (GHTM), Instituto de Higiene e Medicina Tropical (IHMT), Universidade Nova de Lisboa (UNL), 1349-008 Lisbon, Portugal; anareis@ihmt.unl.pt (A.S.-R.); filomenofortes@ihmt.unl.pt (F.F.); 3 Health and Technology Research Center (H&TRC), Escola Superior de Tecnologia da Saúde de Lisboa, Instituto Politécnico de Lisboa, 1990-096 Lisbon, Portugal; 4 Faculdade de Medicina da Universidade Agostinho Neto, Luanda, Angola; 5 Centro de Estatística e Aplicações da Universidade de Lisboa (CEAUL), Faculdade de Ciências da Universidade de Lisboa, 1749-016 Lisbon, Portugal

**Keywords:** stunting, Z-score, growth, longitudinal, deworming, intestinal parasites

## Abstract

Malnutrition and intestinal parasites continue to have serious impacts on growth and cognitive development of children in Angola. A longitudinal four-arm randomized parallel trial was conducted to investigate if deworming with a single annual dose of albendazole (annual-ALB) or a four-monthly test-and-treat (4TT) intestinal parasites approach at individual or household levels improve nutritional outcomes of pre-school children in Bengo province. Children with intestinal parasites (n = 121) were randomly assigned (1:1:1:1) to arm A1: annual-ALB*individual level; A2: annual-ALB*household level; A3: 4TT*individual; and A4: 4TT*household level. At baseline, 4, 8, 12, 16, 20, and 24 months of follow-up, growth was assessed by height, weight, height-for-age, weight-for-height, weight-for-age, and mid-upper arm circumference. Intention-to-treat analysis was done using non-parametric approach, mixed effect models, and generalized estimating equations (GEE). Initially, 57% and 26% of the children were infected by *Giardia lamblia* and *Ascaris lumbricoides*, respectively. This study did not show that a 4TT intestinal parasites approach results on better growth outcomes of children (height, weight, HAZ, WAZ, WHZ and MUACZ) when compared with annual ALB, with exception of height and WHZ using GEE model at 5% level. Positive temporal effects on most nutrition outcomes were observed. Implementing a longitudinal study in a poor setting is challenging and larger sample sizes and ‘pure and clean’ data are difficult to obtain. Nevertheless, learned lessons from this intensive study may contribute to future scientific research and to tailor multidisciplinary approaches to minimize malnutrition and infections in resource-poor countries.

## 1. Introduction

Undernutrition is a major public health concern in developing countries contributing 45% of all child deaths worldwide and to high levels of morbidity [1]. Stunting, or chronic undernutrition, is an indicator of linear growth retardation with a negative impact on child health and educational performance and, in a broader perspective, on economic development and poverty of nations [1,2]. Globally, 149 million children under-five were stunted, and the second Sustainable Development Goal calls for ending malnutrition by 2030 [1,3]. A recent study revealed that many low- and middle-income countries (LMICs) remain far from the World Health Organization Global Nutrition Targets to reduce stunting and wasting by 2025. Sub-Saharan Africa is the region with the shortest children between 2–5 years, after South Asia, while the tallest are in Europe and Central Asia, revealing an uneven distribution around the world [4,5]. 

Malnutrition can result from exposure to poor nutrition, reduced access to healthcare services, inadequate water and sanitation, and recurrent infections [1]. At the same time, intestinal parasitic infections are also a common public health problem in tropical regions, especially in developing regions where defecation habits, poor hygiene, and living conditions can increase the risk of infection [6]. Soil-transmitted helminths (STH) infections (caused by *Ascaris lumbricoides*, *Trichuris trichiura*, and the hookworms *Necator americanus* and *Ancylostoma duodenale*) and intestinal protozoa (such as *Giardia lamblia* and *Cryptosporidium* spp.,) have been also associated to impaired growth [7,8,9]. In STH endemic areas (prevalence ≥ 20.0%), the World Health Organization (WHO) recommends deworming with albendazole (ALB) or mebendazole in pre-school age children (PSAC) and school-age children (SAC) [7]. Other preventive strategies include access to improved water, sanitation, and hygiene conditions.

Angola is a sub-Saharan country that faced a 40-year period of war until 2002 with massive destruction of infrastructures, forced migration, increased malnutrition, diseases and deaths [10]. Ten years later (in 2012), the government made a commitment to reducing stunting and wasting to less than 5% and underweight to less than 10% in children under-five years until 2021 [11]. However, according to the Multiple Indicator Health Survey (MICS) 2015–2016, a large proportion of children are still suffering from stunting (37.6%) and wasting (4.9%) [12]. In Bengo province, the smallest in the country, undernutrition was previously reported as one of the major causes of death in children under five between 2009 and 2012 [13]. Additionally, STH were also commonly reported in PSAC (22.3%), SAC (31.6%), and mothers (28.0%) at community level [14], while rotavirus (25.1%), *Cryptosporidium* spp. (30.0%), and *Giardia lamblia* (21.6%) were reported as the most frequent agents of diarrhea in PSAC attending the referral hospital for the province [15]. At national level, preventive chemotherapy with albendazole (ALB) in SAC, along with access to better hygiene and water, is the strategy to reduce morbidity caused by STH infections [16].

Although preventive chemotherapy has been shown to impact overall STH prevalence levels in a variety of countries, there is a scientific debate on the health benefits and cost-effectiveness of deworming [17]. Some studies, including the Cochrane Systematic Review published in 2019, argue that there are no benefits on growth and hemoglobin levels after deworming children at the community level (infected and non-infected) living in endemic areas for STH [18,19,20]. Similarly, no significant effect of routine deworming was found on weight gain and on mortality of two million PSAC in India [21]. Conversely, analysis of more than 320,000 PSAC from 45 countries found that those who were dewormed were less likely to be stunted and anemic [22]. Results are controversial and it is clear that further research with new approaches is needed [23].

This longitudinal study was implemented to investigate if treatment of intestinal parasites (with or without previous diagnosis) in two levels (individual or household) impacts on nutritional status of children 2–5 years old, after a two-year follow-up. Conducting research at the individual versus household level is important to understand if including all household members can benefit child growth by preventing transmission among family. Moreover, we considered the inclusion of children older than 24 months of age, not only because this is the age at which children become more frequently infected with intestinal parasites, but also to investigate the contribution of these different strategies in reducing chronic malnutrition beyond the well-known “window of opportunity” [4].

## 2. Materials and Methods

### 2.1. Study Design, Setting, and Participants

This four-arm randomized parallel trial was conducted in the Dande Health and Demographic Surveillance System (HDSS) study area, implemented by the Health Research Centre of Angola (CISA), which includes Caxito, Mabubas, Úcua, and a small part of Kikabo communes, located in Dande municipality, Bengo province, Angola [24]. Enrolment was conducted between December 2013 and December 2014 at three outpatient health units in Caxito: Hospital Geral do Bengo, Hospital Municipal do Dande, and Posto Médico o Bom Samaritano.

**Figure 1:**
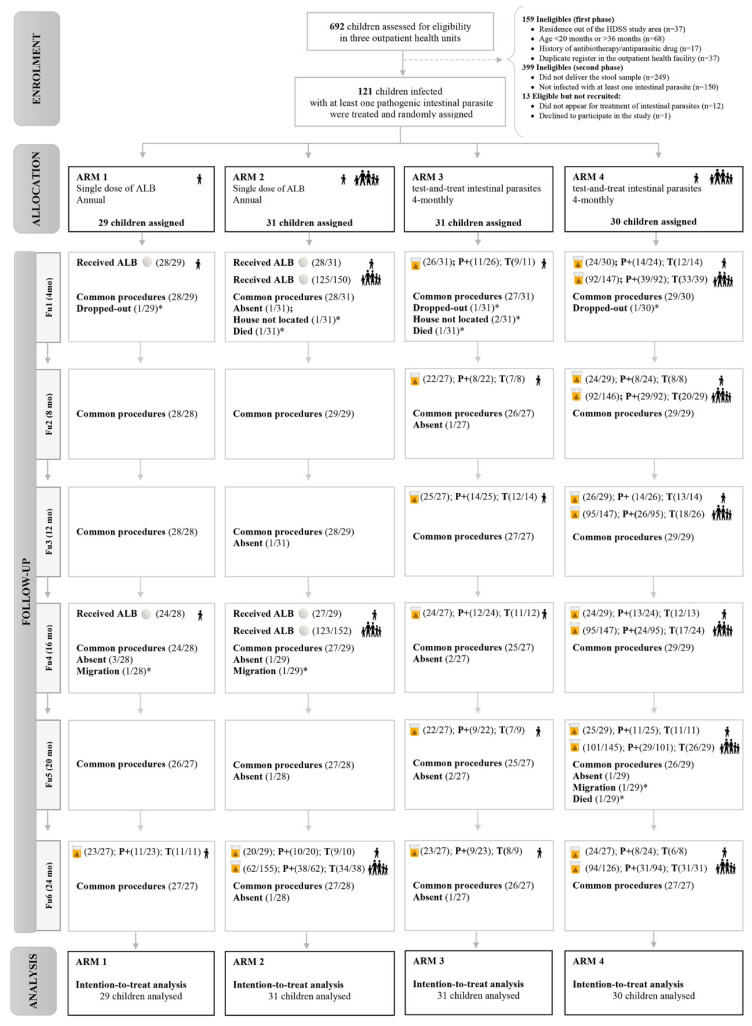
CONSORT flow diagram of the progress through the phases of the four-arm parallel randomized trial (enrolment, intervention allocation, follow-up, and data analysis). Common procedures include anthropometric measurements (weight, height, MUAC), and clinical questionnaire on symptoms, haemoglobin assessment and malaria diagnosis. * Excluded from the following follow-up; mo: months after allocation; 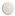: Single dose of albendazole; 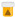: Stool sample collected; P+: Positive samples for pathogenic intestinal parasites; T: children infected with intestinal parasites who received treatment; 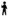: children included; 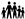: household members.

At baseline, a questionnaire was applied in Portuguese to collect sociodemographic information, clinical condition, and medication history. Anthropometric measurements of weight, height, and mid-upper arm circumference (MUAC) were assessed. A single blood sample was collected for anemia and malaria diagnosis, and a stool sample per child was requested for diagnosis of intestinal parasites. In a first phase, children were deemed eligible according to inclusion criteria: age between 20–36 months at the recruitment (so after four months, in the first follow-up, children were at least 24 months of age; and at the end of study, after two years, children were no more than 59 months of age); residence in the HDSS area; and no history of antibiotic or antiparasitic drug (previous 10 days). Then, if an infection with at least one pathogenic intestinal parasite was found, the child met all criteria to proceed in the study and was randomly allocated to one of four arms (for more detail see Section 2.7—Figure 1). Household members were also included in two of the four arms (Arms 2 and 4). At baseline (before randomization and interventions allocation), all children were treated according to the parasitological diagnosis and considering the standard treatment protocol (Table A1 in the Appendix A). 

Exclusion criteria was applied for children who did not appear to receive medication at baseline or whose parents did not consent their participation in the study. For household members, exclusion criteria included any person who did not live in the same house of the child, and those who refused to participate in the study. 

### 2.2. Randomization and Masking

Children were assigned (1:1:1:1) by a nurse using a simple randomization process (a number was generated from s set {1,2,3,4}, according to a discrete uniform distribution with probability 0.25) to one of the four arms:

- Arm 1 (A1)—to receive a single annual dose of ALB 400 mg at individual level.
- Arm 2 (A2)—to receive a single annual dose of ALB 400 mg at household level (child and household members).
- Arm 3 (A3)—to test-and-treat pathogenic intestinal parasites every four months at individual level.
- Arm 4 (A4)—to test-and-treat pathogenic intestinal parasites every four months at household level (child and household members).

### 2.3. Common Procedures between Arms

Community follow-up (Fu) was performed at 4 (Fu1), 8 (Fu2), 12 (Fu3), 16 (Fu4), 20th (Fu5), and 24 months (Fu6) after inclusion of each child to collect information on reported symptoms, anthropometric measurements, anemia, and malaria diagnosis. 

### 2.4. Symptoms

Reported symptoms were related to the previous week and included fever, vomiting, blood in the stool, and diarrhea (defined as three or more loose or liquid stools per day). 

### 2.5. Anthropometric Assessment

Anthropometric measurements including weight (using scale seca^®^877), height (using seca^®^213, with precision of 0.1 cm), and MUAC were assessed by trained health professionals. MUAC values were transformed to standardized Z-score (MUACZ) using WHO ANTHRO Software (version 3.2.2) (WHO, Geneva, Switzerland) [25]. Weight and height measurements were converted to anthropometric indices in Z-scores: height-for-age (HAZ), weight-for-height (WHZ), and weight-for-age (WAZ) [25]. HAZ was used to identify stunting (or chronic malnutrition) since it reflects cumulative linear growth as a result of recurrent nutrition deprivation and infections [26]. WHZ was used to identify wasting (or acute malnutrition) as it can represent weight loss due to recent episode of infection or inadequate nutrient intake [25]; WAZ, in turn, was calculated to identify children with underweight, which can be a consequence of both acute and chronic malnutrition. According to 2006 WHO Child Growth Standards, a Z-score below −2 for HAZ, WHZ, and WHZ classifies a child as stunted, wasted or underweight, respectively, which includes the moderate (−3 ≤ Z-score < −2) and the severe forms (Z-score < −3) [25]. In addition, children included in this study were also classified as mildly malnourished (−2 ≤ Z-score < −1) and eutrophic (Z-score ≥ −1). Children with clinical symptoms of bilateral pitting edema were classified as acute severely malnourished [27]. 

### 2.6. Anemia and Malaria

A blood sample collected by finger prick was used to determine the hemoglobin (Hb) concentration using the HemoCue^®^ Hb 301 System (HemoCue^®^ AB, Angelhome, Sweden) and to classify anemia as: no anemia (≥11.0 g/dL), mild: (10.0—10.9 g/dL), moderate (7.0—9.9 g/dL), and severe anemia (<7.0 g/dL). Malaria diagnosis was performed by rapid immunochromatographic test (Standard Diagnostics Bioline Malaria Ag *P.f/P.v*, Standard Diagnostics Inc., Giheung-gu, Korea).

### 2.7. Interventions

Figure 1 presents the Consolidated Standards of Reporting Trials (CONSORT) flow diagram of the progress through the phases of the four-arm parallel randomized trial (enrolment, intervention allocation, follow-up, and data analysis). Here, we detail the interventions.

#### 2.7.1. Annual Single Dose of ALB at Individual (A1) and Household Levels (A2)

Annual single dose of ALB 400 mg was performed in children included in A1 and A2. In other words, it means that these children received a single dose of ALB twice during the follow-up period: one dosage four months after inclusion (Fu1) and the second one a year later (16 months after inclusion, Fu4)—Figure 1. In both cases ALB was provided without any knowledge of current infection status. In A2, ALB was also administered at household level: household members between 12 months and 24 months received a single dose of ALB 200 mg, while those older than 24 months received a single dose of ALB 400 mg. At the end of the study, an additional stool sample was requested from participants in each arm for the diagnosis and treatment of intestinal parasite. We considered that it was ethically more appropriate, and it would allow us to understand the pattern of infection among participants who received ALB during the follow-up. This did not influence the comparison of outcomes between the four arms since they were assessed before requiring the stool sample.

#### 2.7.2. Test-and-Treat Intestinal Parasites Approach at Individual (A3) and Household Levels (A4)

Test-and-treat intestinal parasites (from Fu1 to Fu6) was performed every four months in A3 and A4 at individual and household levels, respectively (Figure 1). A stool sample per child (in A3) or per household member (in A4) was requested for microscopic detection of intestinal parasites using direct saline and iodine mounts, formalin-ether sedimentation technique (Parasite Recovery System, PRSTM, Alphatec, Vancouver, WA, USA), modified Ziehl-Neelsen technique (for the identification of *Cryptosporidium* spp., *Isospora belli* and *Cyclospora cayetanensis* oocysts), and Kato–Katz smears (for the quantitative diagnosis of intestinal schistosomiasis and STHs within the 60 min of slide preparation Vertergaard Frandsen, Switzerland) [28]. If *Entamoeba histolytica/dispar* was identified through microscopy, a rapid test for the qualitative detection of *Entamoeba histolytica* was also performed (TECHLAB^®^ E. HISTOLYTICA QUICK CHECKTM, code T30409, Blacksburg, VA, USA).

Microscopic analysis was carried out by two blinded microscopists, and by a third one for discordant results. Participants with positive results received medication by clinical staff, in Hospital Geral do Bengo, and according to a clinical protocol (Table A1 in the Appendix A). An additional stool sample was requested 10 days after completing the standard treatment to ensure its effectiveness. Children younger than 12 months, pregnant women, and women breastfeeding did not receive any of the four interventions [29].

### 2.8. Outcomes

The main outcomes were height, weight, HAZ, WHZ, WAZ, and MUACZ, from baseline to 4, 8, 12, 16, 20, and 24 months of follow-up to assess growth. HAZ was considered the primary outcome because it is an indicator for monitoring infant and young child malnutrition and using it instead of WHZ is easier since weight is sensitive to fluctuations over time [30]. Other secondary outcomes included the occurrence of infection by intestinal protozoa and helminths in all follow-up assessments in A3 and A4. 

### 2.9. Statistical Analysis

#### 2.9.1. Sample Size Calculation

**Figure A1:**
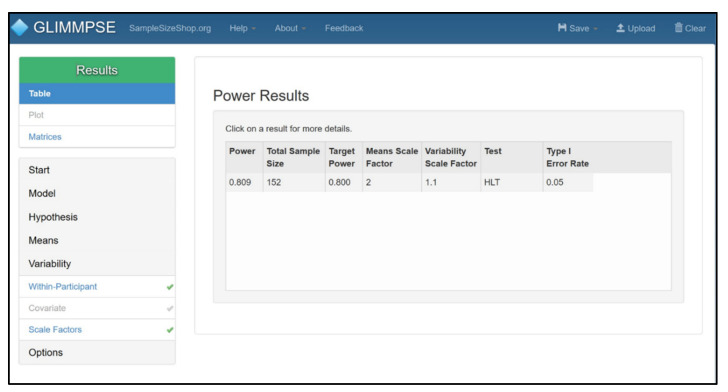
GLIMMPSE software, version 2 (https://glimmpse.samplesizeshop.org/#/solvingFor) (last accessed on: 1 December 2020).

Sample size calculations of this four-arm experimental study with seven repeated measures (baseline, 4, 8, 12, 16, 20, and 24 months of follow-up) were explored in G*Power and GLIMMPSE software (GNU General Public License, version v2). The last one permits more realistic options in terms of the correlation structures among measures. The sample size was based on the primary outcome HAZ [30]. A total of 152 participants with intestinal parasitic infections (38 per arm) were indicated to achieve a power of 80% to test the arm effect, a type error 1 of 0.05, and applying the Hotelling–Lawley Trace test, and a correlation matrix with a decreasing correlation for farther time periods (Figure A1 in the Appendix B). 

#### 2.9.2. Data Analysis

According to CONSORT guidelines, intention-to-treat (ITT) analysis is widely recommended to avoid bias associated with non-random loss, preserving the benefit of randomization [31]. Thus, all randomized participants were included, after a missing values treatment [32]. Analyses to main outcomes started describing the proportion of subjects with missing values by arms and choosing different methods to handle missing data. Special attention was given to height due to facility in terms of interpretation, since height increases over time and this was a way of ensuring that imputation for missing data was performed as close as possible to reality. In cases where a missing value was flanked by valid observations, interpolation was used to height, since there is a monotonic increasing in their values. For the remaining values, we performed multiple imputation through IBM SPSS Software, version 24, using the Expectation Maximization algorithm, identifying the most plausible mechanism underlying our data. Missing Completely at Random (MCAR) was tested using Little’s test [33]. Baseline characteristics such as age and sex, and anthropometric measures assessed at each time point for the corresponding variable of interest were included in the imputation model. Five imputed datasets were computed first for height and then for weight and MUAC. The imputation model performance was also checked for all variables.

After an exploratory analysis and hypothesis tests, Student’s *t*-test was applied to compare the means of two independent groups, and ANOVA to compare the means of more than two groups. When requirements for the independent samples were not met (homogeneity of variances checked by Levene test and normality by Shapiro–Wilk test), nonparametric Mann–Whitney–Wilcoxon and Kruskall–Wallis were used instead. Paired *t*-test or Wilcoxon or Friedman tests were used to compare two or more moments. McNemar and Q-Cochran tests were applied for paired binary variables in two or more time points. 

Multivariate analysis was explored to determine nutritional changes induced by interventions throughout the follow-up assessments. Given the failure of the assumptions of the classic repeated measures, non-parametric approaches were used for quantitative variables [34]. Initially, nonparametric rank-based methods were explored in the *nparLD* (R program) to address the key questions for each continuous outcomes [35]:

(i) Do the arms/treatments have the same effect?
(ii) Is the time profile flat or there is a trend over the follow-up period?
(iii) Are the effects of the treatments similar over time?

This rank-based approach is robust to outliers and present a competitive performance for small sample sizes [35]. However, other strategies are advantageous [32,36]. Thus, linear mixed effect models (LMM) and generalized estimating equations (GEE) for longitudinal data were explored to reinforce our findings, using *lme4, nlme*, and *geepack* packages [32,36]. Different correlation structures were considered in several LMM and GEE models. Plots were explored using gglopt2 package. Initial data analysis was done using IBM SPSS Software, version 24 (IBM Corp, Armonk, NY, USA), and advanced modelling using R Program (R Core Team, Vienna, Austria).

## 3. Results

Between December 2013 and December 2014, 692 children were assessed for eligibility, of which 121 were included and randomly assigned to one of the four arms for two years of community follow-up completed in January 2017. In addition, approximately 150 household members in A2 were also assessed for deworming in two different times (Fu1 and Fu4); while in A4 an average of 143 members per follow-up were assessed for diagnosis and treatment of pathogenic intestinal parasites (Figure 1).

### 3.1. Baseline Characteristics

Table 1 shows baseline sociodemographic characteristics by study arms. The mean age (± standard deviation) of overall children was 26.6 ± 4.86 months and 50.4% of them were female. Considering the sex distribution by arm, more than half of children from arms A1 and A2 were male (55.2% and 58.1%, respectively). Female children were predominant in A3 and A4 (58.1% and 56.7%). The average age of children was slightly lower in A1 and A3 than in A2 and A4. Overall, the proportion of mothers without any education level 11/116 (9.5%) was higher compared with fathers 2/115 (1.7%), *p* = 0.04. The distribution of children, considering parents’ age, their education level, and living conditions was well balanced across arms, as expected in a randomized trial. The river, which is an unimproved drinking water source, was commonly used as a resource water for drinking and/or bathing. Besides, it was also observed that 19.7% of overall children lived in households without sanitation facilities (Table 1). 

At baseline, overall children had mean values of height (84.63 cm ± 5.0) weight (11.26 kg ± 1.79), HAZ (−1.34 ± 1.33); WHZ (−0.29 ± 1.18); WAZ (−0.80 ± 0.98), and MUACZ (−0.88 ± 0.98). As described in Table 2, anthropometric indices were similar across arms and on average all negative, with exception of WHZ in A3. The percentage of stunted children across arms was higher than those with wasting or underweight (Table 2). 

The great proportion of children was classified as having mild malnutrition compared to moderate-to-severe forms, regardless of being mild stunting (27.6% in A1, 32.3% in A2, 38.7% in A3 and 33.3% in A4), mild wasting (A1: 27.6%; A2: 19.4%; A3: 19.4%; A4: 20.0%), or mild underweight (A1:24.1%; A2: 40.0%; A3: 22.6%; A4: 31.0%) (Table 2). Regarding intestinal infections, monoparasitism was more common in all arms than polyparasitism (the presence of two or more pathogenic intestinal parasites—Table 2). 

Children were more frequently infected by protozoa infections (single or multiple), with exception of those in A4 where the percentage of helminths was slightly higher (56.7% versus 53.3%). *Giardia lamblia* was the most common pathogenic agent, accounting for at least 50% of infection in children from the four arms. Other parasitic infections included also other enteric protozoa, such as *Cryptosporidium* spp. and *Entamoeba histolytica*; and helminths including *Strongyloides stercoralis*, *Ascaris lumbricoides*, *Hymenolepis nana*, and *Trichuris trichiura*. Of the 31 *A. lumbricoides* positive samples, Kato–Katz was performed in 17 samples: 11 (64.7%) with light intensity and six (35.3%) with moderate-to-severe intensity. Similarly, *T. trichiura* infections were mainly of light intensity (Table 2).

Overall, malaria was diagnosed in 11 children (9.1%) at baseline. Fever was the most frequently reported symptom (82.8% in A1, 80.6% in A2; 71.0% in A3, and 86.7%), followed by diarrhea (51.7%, 32.3%, 51.6%, and 56.7%, respectively) and vomiting (13.8%, 22.6%, 16.1%, and 6.7%, respectively). Overall, more than 50% of children presented mild-to-severe anemia (Table 2).

### 3.2. Loss to Follow-Up and Missing Values

Of the total, 12 (9.9%) children were permanently lost to the follow-up and did not perform the following assessment due to death (3), house not located (3), dropped-out (3), and emigration (3/12). Temporary withdrawal occurred in children from all arms (Figure 1 and Appendix C). A total of 96 (79.3%) children concluded the study with complete data. For the remaining 25 children (20.7%), there was at least one missing value during the follow-up. No differences in terms of missing values were found among arms (*p* = 0.534). Table A2 in the Appendix D presents complete outcomes and missing values by arms. Little’s test suggests a MCAR missing pattern.

### 3.3. Effect of Interventions on Nutritional Outcomes

The primary analysis was ITT and involved all patients randomly assigned. Table A3 (See Appendix E) shows that, on average, children from A1 (younger) had lower stature and weight at entry and persisted, however, no significant differences were found comparing the four arms. Considering mean values of HAZ, WHZ, and WAZ, no differences were detected among arms in any follow-up assessment. Despite a slight improvement, mean values of HAZ remained negative and far from zero in all six moments (ranging from −1.42 ± 1.19 to −0.99 ± 0.98). Negative values also persisted for WAZ (−1.00 ± 1.03 to −0.57 ± 0.85), and MUACZ (−0.95 ± 0.85 to −0.59 ± 0.82), whereas positive mean values were registered for WHZ (ranging from −0.51 ± 1.06 to 0.14 ± 0.97).

During the follow-up period, moderate-to-severe stunting in children varied between 19.4% and 36.7%, while mild-to-severe stunting ranged from 44.8% to 72.4% (values presented in bold in Table A4—Appendix E). Until the end of the study, a significant decrease was observed in mild-to-severe stunting (mainly 27.6% in A1, 19.3% in A2 and 20.0% in A4), but not in moderate-to-severe stunting (although in A2 the reduction from 35.5% to 19.4% was close to be significant). Analyzing the progress of stunting separated by sex, no significant differences were observed among arms and between the first and the sixth follow-ups (Table A5—Appendix E). Moreover, no differences between the initial and final prevalence of wasting and underweight were also registered within arms (Table A4—Appendix E).

Regarding the three key questions (i, ii, and iii), analyzing simultaneously the effects of arm (treatment), time (follow-up) and interaction arm*time on anthropometric outcomes, findings of *nparLD*, LMM and GEE models are shown in Table 3. 

According to *nparLD*, no significant arm effects were found nor by arm*time interaction. However, temporal changes (effect of time) occurred in all nutritional outcomes (*p* < 0.05) (Table 3). Exploring the same questions using LMM and GEE models, similar patterns were observed. Nevertheless, GEE models indicated a significant effect of arm intervention on height (*p* = 0.02) and WHZ (*p* = 0.04) (Table 3). 

Considering A1 as a reference, parameter estimations associated to GEE and LMM models are presented in Table 4. The results were very similar, even for different correlation structures, enhancing significant temporal changes in almost outcomes (except WAZ for GEE and LMM models and MUACZ for GEE model). 

Regarding to GEE analysis, for height, based on population-average, children from A2 are estimated to have 2.1 cm (SE = 1.32) more than children from A1, while those from A4 and A3 are estimated to have 1.4 cm (SE = 1.44) and 0.3 (SE = 1.26) more than children from A1, respectively, Table 4. These estimates were similar to parameter estimation obtained in LMM models. In terms of height, an increase of 2.5 cm is expected per four-months.

**Figure 2:**
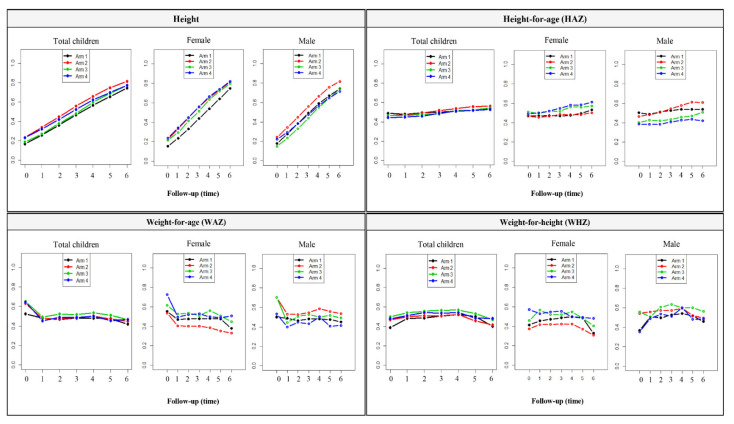
Estimates of the relative effects of arm over time for outcomes to overall and by gender (*nparLD*).

Figure 2 shows the estimates of the relative treatment effects (RTE) of each arm over time obtained by the rank-based approach for some of the outcomes (for overall and by sex). Plots from *nparLD* approach depicts an increase in the effect of height over time, as expected. For HAZ, an almost overlap of lines is visible over time. By gender, plots show a change in the trajectories of HAZ and WHZ, at least in Fu5 and Fu6 (Figure 2). In terms of WAZ and WHZ, from Fu4 to Fu6, a decrease was observed, particularly in A2. By sex, also without significance differences, A2 presented lower values in females, namely for WAZ and WHZ.

### 3.4. Effect of Test-and-Treat Intestinal Parasites Approach on Secondary Outcomes

**Figure 3:**
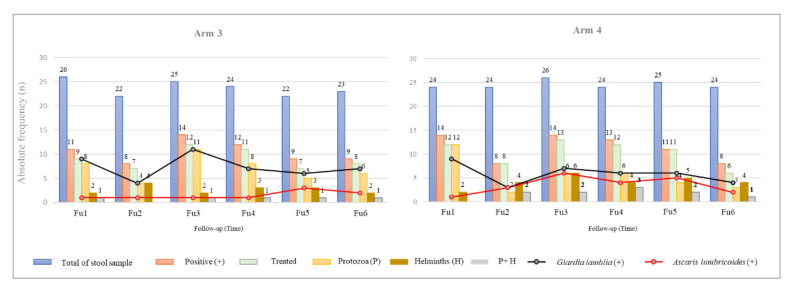
Diagnosis and treatment of intestinal parasites in children allocated to A3 and A4 (four-monthly test-and-treat approach).

From Fu1 to Fu6, there was a significant reduction of overall infection in A4 (*p* = 0.039), but not in A3 (*p* = 0.727). The proportion of infected children slightly fluctuated throughout the study period in both arms. As shown in Figure 3, there was no significant reduction in either *Giardia* or *Ascaris* infection in A3 or A4 (for a more detailed description of data, see the Appendix F).

Comparing the four arms at the end of the study, higher frequencies of infection by *G. lamblia* were identified in children from A2 (40%), followed by A1 (34.8%), A3 (30.4%), and A4 (16.7%), whereas infections caused by *A. lumbricoides* remained similar in children from all arms (near 9% for A1 and A2, 10% in A3, and 8% in A4).

## 4. Discussion

In this study, we assessed the effect of treating intestinal parasites (including protozoa and helminths) diagnosed over time on the nutritional status of children (arms A3 and A4), besides giving ALB without knowing the current infection status (arms A1 and A2).

### 4.1. Malnutrition Is a Public Health Problem in Bengo

A very high proportion of stunting was found among overall children in this study (30.6%, ranging from 25.9% to 33.9% among arms), similar to the estimates for Africa (30.0%) [3], but lower than the national (37.6%) and Bengo (39.7%) prevalence levels previously reported [12]. This was probably because the majority of participants was from urban areas, known to have lower prevalence of stunting compared with rural areas [12]. The percentage of wasting in overall children was 7.4%, very close to the Africa region estimates (7.1%), but higher when compared to MICS prevalence levels (4.7% for Bengo and 4.9% for the country-level) [3,12]. This was possibly because children were recruited in health units seeking for healthcare services instead of being recruited from the community. As expected, poor household conditions were observed, considering sanitation, water sources for drinking, type of construction, and parents’ education level.

### 4.2. Interventions and Nutrition Outcomes of Children between 24 and 60 Months of Age

Assessing child growth is important for detecting deviations from standard references and identifying the effectiveness of planned interventions (Mercedes de Onis et al., 2012). At baseline, children were on average 26.6 ± 4.86 months of age, with mean value of HAZ (−1.34 ± 1.33) and WHZ (−0.29 ± 1.18) similar to the pattern described in PSAC from 57 countries including also sub-Saharan African countries (−1.43 ± 1.70 and −0.05 ± 1.52, respectively) [37]. During the study, mean values of HAZ remained negative and far from zero in all six moments (ranging from −1.42 ± 1.19 to −0.99 ± 0.98). 

Results from this longitudinal study suggested that 4TT intestinal parasites approach did not show better growth outcomes of children (height, weight, HAZ, WAZ, WHZ, and MUACZ) when compared with annual ALB, with exception of height and WHZ using GEE model at the 5% level. According to *nparLD*, the effect of time was significant for all main outcomes. However, temporal changes on WAZ were not significant for GEE and LMM, as well on MUACZ for GEE model. LMM and GEE models estimated an expected four-monthly increase of 0.04 in HAZ (≈0.01 per month), slightly higher than the catch-up growth reported for African children older than 24 months (0.005 z-score per month) [4]. 

Although no differences were detected between interventions, this does not mean that children have not benefited from the effects of interventions. HAZ mean values reported in other African countries such as Congo, Zambia, Namibia, Cameron, and Côte D’Ivoire exhibited some fluctuations [4]. Our children of 24, 36, 48, and 59 months of age appear to be better than the HAZ mean values of the mentioned countries (see Figure A3 in the Appendix G). Growth faltering has been reported in low- and middle-income countries where children are already born with mean values of HAZ below the WHO reference and continue to decrease substantially until 24 months of age, after which it increases slightly until 60 months of age [4]. In our study, HAZ mean values of overall children considered by age group (Figure A3—Appendix G) were higher than the national values reported in 2015/2016 for Angolan children: HAZ score of −1.9 for children between 24–35 months; −1.7 for children between 36–47 months of age; and −1.4 for those between 48–59 months of age [12].

### 4.3. Blind Deworming Neglects Other Parasitic Infections Contributing to Malnutrition

ALB is a well-tolerated anthelminthic drug used for deworming (or preventive chemotherapy), as recommended by WHO [7]. However, studies show that a single dose regimen of ALB has low efficacy against *T. trichiura* (cure rate of 28%) and longer-regimens would be needed to a higher efficacy against *T. trichiura* (single dose of 400 mg/day for 3 days), *S. stercoralis* (oral dose of 400 mg every 12 h for 7 days) and *G. lamblia* (single dose of 400 mg/day for 5 days), previously associated with growth impairment [8,9,38,39,40,41].

This study included children with and without diarrhea, and even for asymptomatic children, *G. lamblia* was the most frequent parasite diagnosed at baseline (57.0%), similar to precedent findings [42,43]. There is evidence that subclinical infection of *G. lamblia* is negatively associated with growth in low resource settings, highlighting the importance of diagnosing and treating the protozoan *G. lamblia* to control the spread of infection, and, consequently, its impact on nutritional status [41]. Thus, it is understandable that giving a single dose of ALB without knowing the current infection status of a child, as performed in arms A1 and A2, may be a disadvantage if the child is infected with giardiasis. Moreover, an increase of intestinal protozoa infections was recently described, including *G. lamblia*, after a five-year period of preventive chemotherapy with a single dose of ALB in SAC in Brazil, despite the reduction in the prevalence of STH, which indicates a change of the epidemiological profile [44].

A higher rate of infection with *A. lumbricoides* was found compared with a previous community-study performed in Bengo (25.6% vs. 15.3%) [14]. However, STH infections were mostly of light intensity and evidence suggests that those who are lightly infected or not infected do not benefit from deworming [7]. This could explain the similar impact of the four treatment on growth in this trial. *Strongyloides stercoralis* was the second most frequent helminth at baseline, highlighting the importance of this neglected STH.

### 4.4. Limitations

First, implementing a longitudinal study in a poor setting is challenging, and not surprisingly our sample was slightly smaller than the sample size calculated, which increases the risk of a false-negative results (Type II error) [45]. 

**Figure A2:**
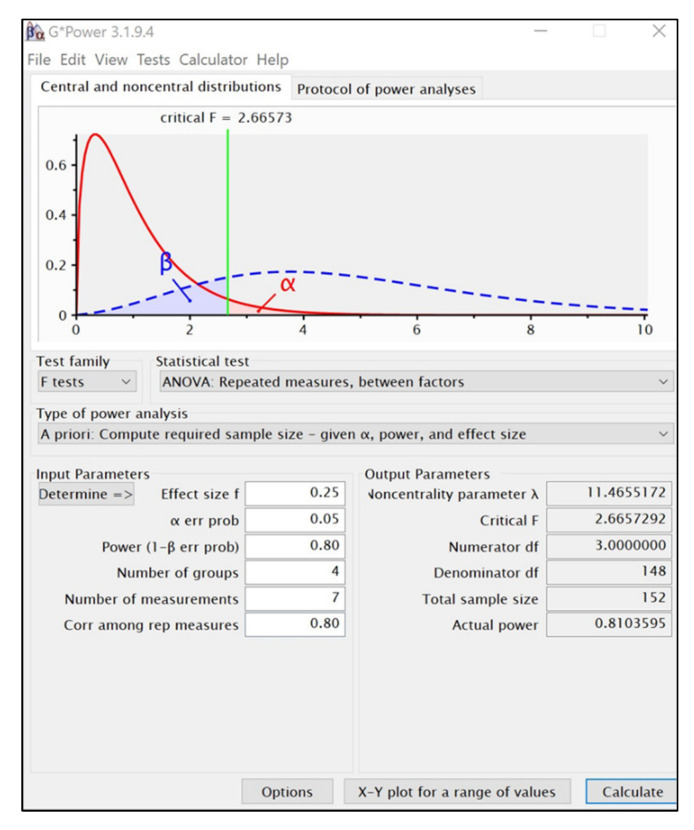
G*Power software, 3.1.9.4 (Heinrich-Heine-Universität Düsseldorf, Düsseldorf, Germany).

**Figure A3:**
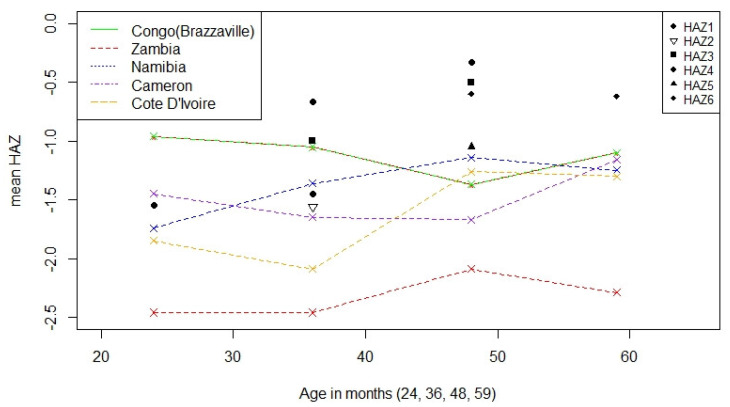
Comparing HAZ mean values from children with 24, 36, 48, and 59 months of age in this study with countries bordering Angola (such as Congo, Zambia, and Namibia) and others lower-middle income countries also with high levels of stunting (Cameron and Côte D’Ivoire.) HAZ scores by country and by age (24, 36, 48, 59 months of age): Congo-Brazzaville (−0.96, −1,05; −1,37; −1.10); Zambia (−2.46, −2.46, −2.09, −2.29); Namibia (−1.74, −1.36, −1.14, −1.25); Cameroon (−1.45, −1.63, −1.67, −1.16); Cote D’Ivoire (−1.85, −2.09, −1.26, −1.30). Data from these countries were obtained from [4].

Second, calculating the design power for this longitudinal four-arm parallel randomized trial considering a continuous outcome was difficult, since the programs are rare and dominated by two groups [46,47,48]. GLIMMPSE and G*Power indicated similar sample size (n = 152), with different assumptions, to obtain a power of 80% to detect differences between arms (Figure A1 and Figure A2 in the Appendix B). For effect of time and the interaction effects, estimated power was 99%, even with 121 children. Of course, 121 children contribute to an underpowered study to our main hypothesis, but this longitudinal study had important benefits for these children and their parents, giving important outputs for future local research. There were several reasons that may have contributed to a smaller sample size obtained in this study, such as a high number not delivering the stool sample during the recruitment period (n = 249), as well as those children who met the inclusion criteria but who did not appear or return at the health unit to be included in the study. Logistically, in resource-poor settings, it is difficult to extend the recruitment for longer periods. The dilemma of not rejecting should not serve as a barrier to the publication of experimental studies, especially of those implemented in low-income countries where research is scarce and population is still fighting against poverty-related health problems such as malnutrition and infectious diseases [49,50]. This study can provide crucial information to the scientific community about its main results, benefits and limitations, and a greater understanding of design issues in developing countries or similar contexts, representing an opportunity to improve field design methods and planning in further research [51]. 

Third, diagnosis of intestinal parasites was performed using a single stool sample in each follow-up, which could have contributed to underestimate the infections. The collection of three stool samples instead of one would be the ideal, but it would represent a logistic burden for caregivers, which could lead to study dropouts and, consequently, compromise the success of follow-up. To overcome this limitation of using a single stool sample per follow-up, different laboratory techniques were performed. 

Fourth, there is no guarantee that participants did not take any other medication. Some treatments in A3 and A4 included more than one dosage/day, and we cannot guarantee that caregivers have complied with the prescription. 

Fifth, self-reported data from questionnaires are also prone to social desirability response bias in any study.

Sixth, adherence to interventions throughout the study was slightly higher in ALB-arms compared with test-and-treat arms, which could have contributed to bias. The greatest difficulty was centered on the delivery of samples, especially when household family members were also included. Follow-up visits can be challenging (for example, the access to some houses was difficult given the poor condition of roads or weather), and it interferes with the dynamics of the individuals, their leisure time, work schedule and responsibilities to the community. In this study, an intention-to-treat analysis was performed according to CONSORT guidelines [31], however, given its design, the study cannot be generalized to the entire population.

### 4.5. Strengths

This trial provides new information about nutrition status of PSAC with intestinal parasitic infections attending three different health units in Bengo province in Angola, and the effect of four different treatment approaches over two years on nutrition outcomes. From a scientific and public health perspective, this is extremely important because there is a need to explore the benefit of other approaches on child growth beyond the preventive chemotherapy with ALB recommended by WHO [20,52]. A study with repeated measurements allows to chart the profile of the same individual across time, an advantage compared with cross-sectional study where different subjects are commonly observed at a specific time point [34]. It is true that missing values can represent a huge challenge in a longitudinal study. However, different methods were applied for imputation and given the fact that data from the same children were collected repeatedly, it decreased the risk of incorrect anthropometric data and subjects can serve as their own controls. Moreover, a study with repeated measurements increases statistical power for detecting changes across time, and smaller sample sizes are needed compared with cross-sectional studies [34]. 

This study contributes with new research approaches by including both intestinal protozoa and helminths when thinking on therapeutic interventions to improve the growth of PSAC. Besides, since individuals living in the same household are exposed to similar risk factors for infection, we have also considered the treatment at household level in A4, and thus, treatment protocol (Table A1 in Appendix A) was designed considering the age, type, and intensity of infection of participants. Screening and treating parasitic infections is more difficult and expensive than preventive chemotherapy, mainly in these settings [53]. In this study, almost 150 household members benefited from deworming with ALB, and 147 members (Fu1), reducing to 126 in Fu6, had free access to diagnosis and treatment of pathogenic intestinal parasites (Figure 1).

From an epidemiological perspective, it is crucial to know the causal agent of an infection in order to plan preventive measures and to provide the access to antimicrobial treatment, especially when children are repeatedly exposed to a wide variety of pathogens. Many research studies are mostly focused on the effect of treating a single pathogen rather than multiple infections. However, in regions where poverty and infections feed the vicious cycle of malnutrition, such as our study setting, the reduced access to adequate water, hygiene, and health conditions contribute to a continuous transmission of multiple pathogens (virus, parasites, and bacteria) [54]. For example, in this setting, rotavirus was previously reported as one of the most important agents causing diarrhea in under-five children [15,55].

Previous studies addressing parasitic infections and malnutrition in Angola were mainly cross-sectional, conducted in SAC, and without any type of longitudinal intervention [14,56,57]. This study includes seven repeated measurements from the same participant (including baseline), which is a key strength of studies with this design. 

The main findings of this study were obtained by three different statistical approaches for longitudinal data, one of them (*nparLD*) described in the literature as robust to outliers and suitable for small sample sizes [35].

Although there was no evidence of significant differences on almost all nutritional outcomes among the four treatment strategies, in order to recommend the best one, an important reduction of mild malnutrition occurred particularly in children from A1, A2, and A4. This is important since all levels of malnutrition, including mild levels, have been previously associated with significantly higher mortality [58]. Repeated and regular community follow-ups for monitoring nutrition outcomes, disease, and providing appropriate treatment may have resulted in indirect benefits on health and wellbeing of participants [59]. Thus, the identification of the pathogenic agents, treatment of infections, healthcare, and informal health promotion provided during two years in the community brought no measurable benefits to children and their families. Moreover, primary data of local and applied research in low-income countries could also help to deal with poverty, diseases, and malnutrition, among others [49], providing important learned lessons to act in a local level and in heterogeneous settings. Although Angola has been at peace since 2002, malnutrition remains a public health problem. Children with mild malnutrition living in precarious environmental conditions, with reduced access to improved hygiene and sanitation conditions and healthcare services, can be at risk of increasing the severity of malnutrition. 

## 5. Conclusions

This longitudinal study suggested that screening and treating intestinal parasites of PSAC, compared with annual ALB (both strategies provided at individual or household level) over two years, provided similar growth outcomes. Additional research is needed to address the effect of these interventions in a longer period and, ideally, in a more heavily infected setting and including a higher number of participants to better understand the benefits of these type of interventions. However, given the cycle of poverty and infection, reducing malnutrition remains a challenge. Given the multiple factors leading to malnutrition, its reduction seems to require multidisciplinary approaches, including maternal and child interventions, safe water and sanitation, access to health care services, food production, availability, and distribution. 

## Figures and Tables

**Table 1:** Baseline sociodemographic characteristics by study arms.

	Variable (n)	Categories ^a^	A1 (n = 29)	A2 (n = 31)	A3 (n = 31)	A4 (n = 30)
**Child**	**Sex** (n = 121)	Male	16 (55.2)	18 (58.1)	13 (41.9)	13 (43.3)
		Female	13 (44.8)	13 (41.9)	18 (58.1)	17 (56.7)
	**Age** (n = 121)	Mean ± SD, (months)	25.1 ± 4.57	27.7 ± 4.93	25.8 ± 4.90	27.6 ± 4.70
	**Exclusively breastfed** (n = 114)	Mean ± SD, (months)	4.6 ± 1.80	5.4 ± 1.82	4.3 ± 2.1	4.7 ± 2.3
	**Complementary feeding** (n = 111)	Mean ± SD, (months)	19.7 ± 3.9	19.6 ± 5.0	20.1 ± 4.1	21.0 ± 3.8
**Mother**	**Age** (n = 114)	Mean ± SD, (years)	27.32 ± 6.44	28.97 ± 6.68	26.67 ± 5.05	30.42 ± 7.90
	**Maternal education** (n = 116)	No education	3 (10.3)	2 (6.9)	0 (0.0)	6 (20.7)
		Primary	8 (27.6)	14 (48.3)	18 (62.1)	11 (37.9)
		Secondary or higher	18 (62.1)	13 (44.8)	11 (37.9)	12 (41.4)
	**Studying and working status** (n = 116)	Do not study/work	5 (17.2)	7 (24.1)	8 (27.6)	5 (17.2)
		Only working	11 (37.9)	8 (27.6)	7 (24.1)	11 (37.9)
		Study and work	4 (13.8)	5 (17.2)	5 (17.2)	5 (17.2)
		Only studying	9 (31.0)	9 (31.0)	9 (31.0)	8 (27.6)
**Father**	**Age** (n = 92)	Mean ± SD, (years)	31.44 ± 7.93	35.07 ± 10.49	30.67 ± 4.90	36.84 ± 10.12
	**Paternal education** (n = 115)	No education	1 (3.4)	1 (3.6)	0 (0.0)	0 (0.0)
		Primary	3 (10.3)	5 (17.9)	6 (20.7)	6 (20.7)
		Secondary or higher	25 (86.2)	22 (78.6)	23 (79.3)	23 (79.3)
	**Studying and working status** (n = 115)	Do not study/work	0 (0.0)	0 (0.0)	1 (3.4)	1 (3.4)
		Only working	21 (72.4)	22 (78.6)	19 (65.5)	23 (793)
		Study and work	6 (20.7)	6 (21.4)	7 (24.1)	4 (13.8)
		Only studying	2 (6.9)	0 (0.0)	2 (6.9)	1 (3.4)
**Household characteristics**	**Members per household** (n = 117)	Mean ± SD	6.29 ± 2.29	6.20 ± 2.30	5.45 ± 1.59	5.97 ± 2.06
	**Place of residence** (n = 121)	Urban	27 (93.1)	31 (100.0)	30 (96.8)	27 (90.0)
	**Rooms** (n = 117)	≤3	23 (79.3)	17 (58.6)	25 (83.3)	22 (75.9)
		>3	6 (20.7)	12 (41.4)	5 (16.7)	7 (24.1)
	**Wall** (n = 117)	Adobe	18 (62.1)	20 (69.0)	23 (76.7)	26 (89.7)
		Bricks	11 (37.9)	9 (31.0)	7 (23.3)	3 (10.3)
	**Floor** (n = 117)	Earth/sand	5 (17.2)	4 (13.8)	3 (10.0)	3 (10.3)
		Cement/ceramic	24 (82.8)	25 (86.2)	27 (90.0)	26 (89.7)
	**Mobile phone** (n = 117)	Yes	27 (93.1)	28 (96.6)	30 (100.0)	25 (86.2)
	**Television** (n = 117)	Yes	25 (86.2)	26 (89.7)	26 (86.7)	26 (89.7)
	**Public electricity** (n = 117)	Yes	21 (72.4)	27 (93.1)	25 (83.3)	21 (72.4)
	**Freezer** (n = 117)	Yes	22 (75.9)	22 (75.9)	23 (76.7)	21 (72.4)
	**Cable TV** (n = 117)	Yes	19 (65.5)	23 (79.3)	20 (66.7)	20 (69.0)
	**Wheelbarrow** (n = 117)	Yes	14 (48.3)	18 (62.1)	16 (53.3)	15 (51.7)
	**Radio** (n = 117)	Yes	16 (55.2)	12 (41.4)	14 (46.7)	13 (44.8)
	**Motorcycle** (n = 117)	Yes	9 (31.0)	9 (31.0)	15 (50.0)	9 (31.0)
	**Generator** (n = 117)	Yes	4 (13.8)	6 (20.7)	8 (26.7)	10 (34.5)
	**Animals** (n = 117)	Yes	6 (20.7)	3 (10.3)	7 (24.1)	7 (24.1)
	**Refrigerator** (n = 117)	Yes	5 (17.2)	4 (13.8)	6 (20.0)	6 (20.7)
	**Car** (n = 117)	Yes	6 (20.7)	4 (13.8)	3 (10.0)	7 (24.1)
	**Bicycle** (n = 117)	Yes	1 (3.4)	2 (6.9)	4 (13.3)	0 (0.0)
**Water source and sanitation**	**Drinking water source ^b^** (n = 117)	Improved	21 (72.4)	22 (75.9)	19 (63.3)	21 (72.4)
		Unimproved	8 (27.6)	7 (24.1)	11 (36.7)	8 (27.6)
	**Drinking water source** (n = 117)	River	8 (27.6)	5 (17.2)	9 (30.0)	7 (24.1)
		Tap in the yard	5 (17.2)	12 (41.4)	7 (23.3)	8 (27.6)
		Private tank	9 (31.0)	6 (20.7)	9 (30.0)	10 (34.5)
		Others ^c^	7 (24.1)	6 (20.6)	5 (16.7)	4 (13.8)
	**Bath water source** (n = 117)	Irrigation channel	9 (31.0)	4 (13.8)	9 (30.0)	6 (20.7)
		River	7 (24.1)	6 (20.7)	9 (30.0)	8 (27.6)
		Tap in the yard	3 (10.3)	7 (24.1)	4 (13.3)	7 (24.1)
		Private tank	3 (10.3)	5 (17.2)	2 (6.7)	4 (13.8)
		Others ^d^	7 (24.1)	7 (24.1)	6 (20.0)	4 (13.8)
	**Latrine** (n = 117)	No facility	7 (24.1)	3 (10.3)	7 (23.3)	6 (20.7)
		Public	10 (34.5)	10 (34.5)	9 (30.0)	7 (24.1)
		Private	12 (41.4)	16 (55.2)	14 (46.7)	16 (55.2)

^a^ For categorical variables characteristics are expressed as n (%); SD means Standard Deviation; ^b^ According to the WHO/UNICEF Joint Monitoring Programme for Water Supply, Sanitation and Hygiene (JMP) [32]. Improved water includes piped water into dwelling, tap in the yard, public tap water, tube well, borehole, covered or uncovered tank, tanker truck; and unimproved water includes river, irrigation channel. ^c^ includes piped water, irrigation channel, borehole and tube well. ^d^ includes piped water, borehole and tube well.

**Table 2:** Baseline nutritional status, infection with intestinal parasites, symptoms reported, malaria, and anemia in children by study arms.

	Variable (n)	Categories ^a^	A1 (n = 29)	A2 (n = 31)	A3 (n = 31)	A4 (n = 30)
**Nutritional status**	**Height** (n = 121)	Mean ± SD	83.63 ± 5.32	85.49 ± 5.89	84.19 ± 5.68	85.15 ± 6.73
	**Weight ^b^** (n = 119)	Mean ± SD	10.70 ± 1.74	11.58 ± 1.77	11.40 ± 1.70	11.48 ± 1.87
	**MUAC** (n = 121)	Mean ± SD	13.83 ± 1.32	14.34 ± 1.12	14.33 ± 1.01	14.40 ± 0.94
	**HAZ** (n = 121)	Mean ± SD	−1.26 ± 1.41	−1.35 ± 1.35	−1.30 ± 1.33	−1.43 ± 1.29
	**WHZ ^b^** (n = 119)	Mean ± SD	−0.65 ± 1.22	−0.25 ± 1.02	0.00 ± 1.36	−0.22 ± 0.98
	**WAZ ^b^** (n = 119)	Mean ± SD	−1.15 ± 1.19	−0.87 ± 1.07	−0.65 ± 1.15	−0.89 ± 1.04
	**MUACZ** (n = 121)	Mean ± SD	−1.19 ± 1.21	−0.85 ± 0.93	−0.73 ± 0.90	−0.75 ± 0.84
	**Stunting** (n = 121)	Eutrophic	12 (41.4)	11 (35.5)	11 (35.5)	10 (33.3)
		Moderate-to-severe	9 (31.0)	10 (32.3)	8 (25.8)	10 (33.3)
	**Wasting** (n = 121)	Eutrophic	17 (58.6)	23 (74.2)	24 (77.4)	22 (73.3)
		Moderate-to-severe	4 (13.8)	2 (6.5)	1 (3.2)	2 (6.7)
	**Underweight** (n = 119)	Eutrophic	16 (55.2)	15 (50.0)	18 (58.1)	16 (55.2)
		Moderate-to-severe	6 (20.7)	3 (10.0)	6 (19.4)	4 (10.3)
**Pathogenic intestinal parasites**	**Type of infection** (n = 121)	Monoparasitism	25 (86.2)	26 (83.9)	28 (90.3)	26 (86.7)
		Polyparasitism	4 (13.8)	5 (16.1)	3 (9.7)	4 (13.3)
	**Group of parasites** (n = 121)	Protozoa (P)	18 (62.1)	17 (54.8)	19 (61.3)	13 (43.3)
		Helminths (H)	8 (27.6)	12 (38.7)	11 (35.5)	14 (46.7)
		P + H	3 (10.3)	2 (6.5)	1 (3.2)	3 (10.0)
	***Giardia lamblia*** (n = 121)	Positive	17 (58.6)	18 (58.1)	19 (61.3)	15 (50.0)
	***Ascaris lumbricoides*** (n = 121)	Positive	3 (10.3)	8 (25.8)	8 (25.8)	12 (40.0)
	***A. lumbricoides* intensity ^c^** (Kato–Katz ^d^, n = 17)	Light	1 (50.0)	4 (66.7)	3 (60.0)	3 (75.0)
		Moderate-to-heavy	1 (50.0)	2 (33.3)	2 (40.0)	1 (25.0)
	***Strongyloides stercoralis*** (n = 121)	Positive	5 (17.2)	3 (9.7)	4 (12.9)	4 (13.3)
	***Trichuris trichiura*** (n = 121)	Positive	2 (6.9)	2 (6.5)	1 (3.2)	2 (6.7)
	***T. trichiura* intensity^e^** (Kato–Katz^d^, n = 3)	Light	1 (100.0)	1 (100.0)	0 (0.0)	1 (100.0)
		Moderate-to-heavy	0 (0.0)	0 (0.0)	0 (0.0)	0 (0.0)
	***Hymenolepis nana*** (n = 121)	Positive	1 (3.4)	3 (9.7)	3 (9.7)	0 (0.0)
	***Cryptosporidium* spp.** (n = 121)	Positive	3 (10.3)	1 (3.2)	1 (3.2)	2 (6.7)
	***Entamoeba histolytica*** (n = 121)	Positive	2 (6.9)	1 (3.2)	0 (0.0)	0 (0.0)
**Malaria, Hb, symptoms**	**Malaria** (*P.falciparum*) (n = 121)	Positive	4 (13.8)	3 (9.7)	2 (6.5)	2 (6.7)
	**Hemoglobin** (g/dL) (n = 116)	mean ± SD	10.3 ± 2.06	10.5 ± 1.65	11.0 ± 1.05	10.5 ± 1.81
	**Anemia** (n = 119)	No	11 (40.7)	15 (48.4)	18 (58.1)	11 (36.7)
		Mild	7 (25.9)	7 (22.6)	9 (29.0)	13 (43.3)
		Moderate	7 (25.9)	7 (22.6)	4 (12.9)	4 (13.3)
		Severe	2 (7.4)	2 (6.5)	0 (0.0)	2 (6.7)
	**Diarrhea** (n = 121)	Yes	15 (51.7)	10 (32.3)	16 (51.6)	17 (56.7)
	**Fever** (n = 121)	Yes	24 (82.8)	25 (80.6)	22 (71.0)	26 (86.7)
	**Vomiting** (n = 121)	Yes	4 (13.8)	7 (22.6)	5 (16.1)	2 (6.7)

^a^ For categorical variables characteristics are expressed as n (%). SD means Standard Deviation; ^b^ Did not include two children with bilateral edema, one from A2 and another one from A4. ^c^ *Ascaris lumbricoides* intensity obtained by Kato–Katz considering the number of eggs per gram of stool (EPG): light intensity infection (1–4.999 EPG); moderate (5.000–49.999 EPG) and heavy (≥50.000 EPG). ^d^ Kato–Katz technique was not performed in diarrhea l stool samples. ^e^ *Trichuris trichiura* intensity obtained by Kato–Katz considering the number of eggs per gram of stool (EPG): light (1–999 EPG); moderate (1.000–9.999 EPG) and heavy (≥10.000EPG).

**Table 3:** ANOVA tables for main outcomes, using a nonparametric analysis of longitudinal data (*nparLD*) and LMM and GEE models.

Outcome	*nparLD*						LMM				GEE		
	ANOVA Modified			Wald			ANOVA				ANOVA		
Effect	Statistic	df	*p*-value	Statistic	df	*p*-value	Statistic	df_1_	df_2_	*p*-value	Statistic ^a^	df	*p*-value
**Height**													
Arm	0.94	2.930	*0.42*	3.03	3	*0.39*	1.0	3	117	*0.48*	10.0	3	0.02*
Time	1210.04	1.903	<0.001 *	1860.84	5	<0.001 *	6997.0	1	601	<0.001 *	427.0	1	<0.001*
Arm*Time	0.79	5.483	*0.57*	11.86	15	*0.69*	0.0	3	601	*0.72*	0.0	3	*1.00*
**Weight**													
Arm	0.63	2.939	*0.59*	1.82	3	*0.61*	1.0	3	117	*0.62*	7.5	3	*0.06*
Time	474.71	3.279	<0.001 *	1053.28	5	<0.001 *	2025.0	1	601	<0.001 *	216.8	1	<0.001*
Arm*Time	0.82	8.979	*0.60*	15.83	15	*0.39*	0.0	3	601	*0.69*	0.1	3	*0.99*
**HAZ**													
Arm	0.06	2.986	*0.98*	0.19	3	*0.98*	0.1	3	117	*0.96*	2.13	3	*0.55*
Time	29.30	3.256	<0.001 *	64.53	5	<0.001 *	54.2	1	601	<0.001 *	5.23	1	0.02*
Arm*Time	0.73	8.670	*0.68*	11.43	15	*0.72*	0.4	3	601	*0.78*	0.13	3	*0.99*
**WAZ**													
Arm	0.12	2.986	*0.95*	0.40	3	*0.94*	0.3	3	117	*0.84*	5.08	3	*0.17*
Time	6.06	4.292	<0.001 *	23.73	5	<0.001 *	1.9	1	601	*0.17*	0.21	1	*0.65*
Arm*Time	0.77	10.112	*0.66*	15.92	15	*0.39*	0.3	3	601	*0.83*	0.04	3	*1.00*
**WHZ**													
Arm	0.29	2.955	*0.83*	0.95	3	*0.81*	0.5	3	117	*0.68*	8.10	3	0.04*
Time	10.15	4.534	<0.001 *	46.32	5	<0.001 *	15.6	1	601	<0.001 *	4.08	1	0.04*
Arm*Time	0.48	11.766	*0.93*	9.63	15	*0.84*	0.06	3	601	*0.98*	0.04	3	*1.00*
**MUACZ**													
Arm	0.35	2.947	*0.79*	1.00	3	*0.80*	0.5	3	117	*0.66*	7.52	3	*0.06*
Time	4.35	4.549	0.001 *	19.15	5	0.002 *	6.8	1	601	0.009 *	1.86	1	*0.17*
Arm*Time	0.40	12.047	*0.96*	6.80	15	*0.96*	1.0	3	601	*0.39*	0.77	3	*0.86*

**^a^** Statistic: χ^2^; HAZ: height-for-age Z-score; WAZ: weight-for-age Z-score; WHZ: Weight-for-height Z-score; MUACZ: Mid-Upper Arm Circumference Z-score; * *p < 0.05.*

**Table 4:** Parameter estimation for outcomes using GEE and LMM models.

Variable	Parameter	LMM					GEE			
		E	SE	Df	Statistic	*p*-Value	E	SE	Statistic	*p*-Value
**Height**	(Intercept)	84.1	1.08	601	78.1	<0.001 *	84.3	0.92	8324.2	<0.001 *
	A1A2	2.1	1.50	117	1.4	0.16	2.1	1.32	2.6	0.11
	A1A3	0.3	1.50	117	0.2	0.85	0.3	1.26	0.1	0.80
	A1A4	1.5	1.51	117	1.0	0.33	1.4	1.44	1.0	0.32
	Time	2.5	0.06	601	40.9	<0.001 *	2.5	0.24	106.67	<0.001 *
	A1A2: Time	0.0	0.09	601	0.0	0.97	0.0	0.34	0.0	0.95
	A1A3: Time	0.1	0.09	601	0.7	0.48	0.1	0.33	0.0	0.85
	A1A4: Time	0.0	0.09	601	−0.4	0.69	0.0	0.38	0.0	0.98
**Weight**	(Intercept)	11.4	0.35	601	32.1	<0.001 *	11.4	0.32	1301.5	<0.001 *
	A1A2	0.6	0.49	117	1.1	0.26	0.6	0.44	1.8	0.18
	A1A3	0.2	0.49	117	0.5	0.62	0.3	0.41	0.4	0.51
	A1A4	0.4	0.50	117	0.8	0.45	0.4	0.46	0.8	0.36
	Time	0.6	0.03	601	21.3	<0.001 *	0.6	0.09	45.1	<0.001 *
	A1A2: Time	0.0	0.04	601	0.2	0.87	0.0	0.12	0.0	0.98
	A1A3: Time	0.0	0.04	601	0.9	0.38	0.0	0.11	0.1	0.81
	A1A4: Time	0.0	0.04	601	1.0	0.34	0.0	0.13	0.0	0.85
**HAZ**	(Intercept)	−1.38	0.210	601	−6.6	<0.001 *	−1.39	0.201	47.7	<0.001 *
	A1A2	0.04	0.292	117	0.1	0.90	0.04	0.278	0.0	0.90
	A1A3	0.00	0.292	117	0.0	0.99	0.01	0.271	0.0	0.98
	A1A4	−0.09	0.295	117	−0.3	0.77	−0.09	0.280	0.1	0.76
	Time	0.04	0.015	601	2.8	0.01 *	0.04	0.051	0.6	0.42
	A1A2: Time	0.02	0.021	601	1.0	0.31	0.03	0.069	0.1	0.72
	A1A3: Time	0.02	0.021	601	0.7	0.46	0.02	0.068	0.1	0.83
	A1A4: Time	0.01	0.021	601	0.6	0.56	0.02	0.071	0.1	0.83
**WHZ**	(Intercept)	−0.08	0.179	601	−0.4	0.67	−0.08	0.181	0.2	0.65
	A1A2	0.06	0.248	117	0.2	0.81	0.08	0.250	0.1	0.75
	A1A3	0.25	0.248	117	1.0	0.32	0.26	0.226	1.3	0.25
	A1A4	0.11	0.250	117	0.4	0.67	0.13	0.240	0.3	0.58
	Time	−0.04	0.022	601	−2.0	0.04 *	−0.04	0.046	0.7	0.41
	A1A2: Time	0.00	0.030	601	−0.1	0.91	−0.01	0.062	0.0	0.87
	A1A3: Time	0.00	0.030	601	0.1	0.94	−0.00	0.058	0.0	0.98
	A1A4: Time	0.01	0.030	601	0.3	0.78	0.00	0.062	0.0	1.00
**WAZ**	(Intercept)	−0.79	0.175	601	−4.5	<0.001 *	−0.80	0.178	20.2	<0.001 *
	A1A2	0.04	0.243	117	0.2	0.86	0.06	0.244	0.1	0.79
	A1A3	0.18	0.243	117	0.7	0.47	0.19	0.228	0.7	0.40
	A1A4	0.01	0.245	117	0.0	0.97	0.04	0.236	0.0	0.88
	Time	−0.02	0.016	601	−1.4	0.16	−0.02	0.046	0.1	0.73
	A1A2: Time	0.02	0.022	601	0.7	0.47	0.01	0.061	0.0	0.88
	A1A3: Time	0.01	0.022	601	0.5	0.65	0.01	0.058	0.0	0.93
	A1A4: Time	0.02	0.022	601	0.9	0.38	0.01	0.060	0.0	0.86
**MUACZ**	(Intercept)	−0.92	0.153	601	−6.0	<0.001 *	−0.92	0.168	30.1	<0.001 *
	A1A2	0.24	0.213	117	1.1	0.27	0.24	0.236	1.0	0.31
	A1A3	0.35	0.213	117	1.7	0.10	0.35	0.120	3.1	0.08
	A1A4	0.36	0.215	117	1.7	0.09	0.36	0.215	2.8	0.09
	Time	0.00	0.021	601	0.2	0.86	0.01	0.042	0.0	0.91
	A1A2: Time	−0.03	0.029	601	−1.1	0.27	−0.03	0.058	0.3	0.57
	A1A3: Time	−0.04	0.029	601	−1.5	0.13	−0.04	0.051	0.7	0.41
	A1A4: Time	−0.04	0.029	601	−1.5	0.13	−0.04	0.054	0.7	0.44

Reference class = A1; E = Estimate; SE = Standard error; * *p* < 0.05.

## Data Availability

The data presented in this study are available on request from the corresponding author. The data are not publicly available due to ethical requirements.

## List of Abbreviations

A1	Arm 1
A2	Arm 2
A3	Arm 3
A4	Arm 4
ALB	Albendazole
Annual-ALB	Single annual dose of albendazole
Annual-ALB*individual level	Single annual dose of albendazole provided at individual level in arm 1
Annual-ALB*household level	Single annual dose of albendazole provided at household level in arm 2
CISA	Health Research Centre of Angola
CONSORT	Consolidated Standards of Reporting Trials
EPG	Eggs per gram
Fu	Follow-up
Fu1	Follow-up performed 4 months after participant inclusion
Fu2	Follow-up performed 8 months after participant inclusion
Fu3	Follow-up performed 12 months after participant inclusion
Fu4	Follow-up performed 16 months after participant inclusion
Fu5	Follow-up performed 20 months after participant inclusion
Fu6	Follow-up performed 24 months after participant inclusion
g/dL	Grams per decilitre
GEE	Generalized estimating equations
HAZ	Height-for-age Z-score
Hb	Hemoglobin
HDSS	Dande Health and Demographic Surveillance System
ITT	Intention-to-treat
LMICs	Low- and middle-income countries
LMM	Linear mixed effect models
MCAR	Missing Completely at Random
MICS	Multiple Indicator Health Survey
MTZ	Metronidazole
MUAC	Mid-upper arm circumference
MUACZ	Mid-Upper Arm Circumference Z-score
*npar*LD	*nonparametric analysis of longitudinal data*
*P.f*	*Plasmodium falciparum*
PSAC	pre-school age children
*P.v*	*Plasmodium vivax*
PZQ	Praziquantel
SAC	School-age children
STH	Soil-transmitted helminths
TNZ	Tinidazole
WAZ	Weight-for-age Z-score
WHO	World Health Organization
WHO/UNICEF JMP	World Health Organization/United Nations International Children’s Emergency Fund Joint Monitoring Programme for Water Supply, Sanitation, and Hygiene
WHZ	Weight-for-height Z-score
4TT	Four-monthly test-and-treat
4TT*individual	A four-monthly test-and-treat intestinal parasites approach provided at individual level in arm 3
4TT*household level	A four-monthly test-and-treat intestinal parasites approach provided at household level in arm 4

## Appendix A. Medication Regimens

**Table A1:** Treatment of pathogenic intestinal parasites provided before interventions allocation and during the follow-ups in Arms 3 and 4.

Type of Infection Intestinal Parasites Identified	12 < Age < 24 Months						Age ≥ 24 Months				
	ALB	MTZ	TNZ	PZQ	Dosage	Frequency	ALB	TNZ	PZQ	Dosage	Frequency
	**Monoparasitism** (single pathogenic intestinal parasite)										
*Ascaris lumbricoides*	x				200 mg	single dose	x			400 mg	single dose
*Ancylostoma* sp.	x				200 mg	single dose	x			400 mg	single dose
*Entamoeba histolytica*		x			35–50 mg/kg/d ^c^	8/8 h, 7d		x		50 mg/kg ^b^	3d
*Giardia lamblia*		x			15 mg/kg/d	8/8 h, 5d		x		50 mg/kg ^b^	single dose
*Hymenolepis nana*				x	25 mg/kg	single dose			x	25 mg/kg	single dose
*Strongyloides stercoralis*	x				200 mg	12/12 h, 7d	x			400 mg	12/12 h, 7d
*Trichuris trichiura*	x				200 mg	3d	x			400 mg	3d
	**Polyparasitism** (at least two pathogenic intestinal parasites)										
*G. lamblia + E. histolytica*		x			35–50 mg/kg/d ^c^	8/8h, 7d		x		50 mg/kg ^b^	3d
*G. lamblia + A. lumbricoides*	x				200 mg	5d	x			400 mg	5d
*G. lamblia + T. trichiura*	x				200 mg	5d	x			400 mg	5d
*G. lamblia + A. lumbricoides + T.trichiura*	x				200 mg	5d	x			400 mg	5d
*G. lamblia + S. stercoralis*	x				200 mg	12/12 h, 7d	x			400 mg	12/12 h, 7d
*G. lamblia + H. nana*				x	25 mg/kg ^a^	single dose			x	25 mg/kg	single dose
		x			15 mg/kg/d	8/8 h, 5d		x		50 mg/kg ^b^	single dose
*A. lumbricoides + S. stercoralis*	x				200 mg	12/12, 7d	x			400 mg	12/12, 7d
*T. trichiura + H. nana*				x	25 mg/kg ^a^	single dose			x	25 mg/kg	single dose
	x				200 mg	3d	x			200 mg	3d

ALB: Albendazole; MTZ: Metronidazole; TNZ: Tinidazole; PZQ: Praziquantel; d: day; ^a^ Praziquantel is administered alone in the first day; ^b^ maximum dose is 2 g; ^c^ maximum dose is 750 mg.

## Appendix C. Losses to Follow-Up

Overall, 12 (9.9%) were permanently lost to the follow-up and did not perform the following assessment. Temporary withdrawal occurred in children from all arms:

- Arm 1:
  ✓ 96.6% (28/29) of children received a single dose of ALB at Fu1;
  ✓ 85.7% (24/28) of children received a single dose of ALB at Fu4
  ✓ Of the total included, 82.8% (24/29) received complete intervention in both Fu1 and Fu4.
- Arm 2:
  ✓ 90.3% (28/31) of children and 83.3% (125/150) of household members received a single dose of ALB at Fu1; Coverage of ALB by household was ≥80% in 25 cases.
  ✓ 93.1% (27/29) of children and 80.9% (123/152) of household members received a single dose of ALB at Fu4; Coverage of ALB by household was ≥80% in 23 cases.
  ✓ 71.0% (22/31) of children received ALB in both Fu1 and Fu4 with a household coverage ≥80%.
- Arm 3:
  ✓ The percentage of children delivering a stool sample and receiving appropriate treatment by follow-up was: 77.4% (24/31) in Fu1; 67.7% (21/31) in Fu2; 74.2% (23/31) in Fu3; 74.2% (23/31) in Fu4; 64.5% (20/31) in Fu5, and 71.0% (22/31) in Fu6.
  ✓ However, only 48.4% (15/31) of children delivered a stool sample and received appropriate treatment in all follow-ups.
- Arm 4:
  ✓ The percentage of children delivering a stool sample and receiving appropriate treatment by follow-up was: 73.3% (22/30) in Fu1; 80.0% (24/30) in Fu2; 83.3% (25/30) in Fu3; 76.7% (23/30) in Fu4; 83.3% (25/30) in Fu5; and 73.3% (22/30) in Fu6.
  ✓ The percentage of household members delivering the requested stool sample during the follow-up period ranged between 63.0% (92/146) and 74.6% (94/126).
  ✓ At the household level, only nine children (30%) and at least 50% of their household members were able to deliver a stool sample and receive appropriate treatment in the six follow-ups.

## Appendix D. Missing Data

**Table A2:** Primary outcomes missing data by study arm.

Follow-Up (2 Years)	Arm 1 (n = 29)	Arm 2 (n = 31)	Arm 3 (n = 31)	Arm 4 (n = 30)	Total (n = 121)
	n (%)	n (%)	n (%)	n (%)	n (%)
No missing values	24 (82.8)	23 (74.2)	23 (74.2)	26 (86.7)	96 (79.3)
1 missing values	2 (6.9)	4 (12.9)	3 (9.7)	1 (3.3)	10 (8.3)
2 missing values	1 (3.4)	2 (6.5)	1 (3.2)	2 (6.7)	6 (5.0)
3 missing values	1 (3.4)	0 (0.0)	0 (0.0)	0 (0.0)	1 (0.8)
6 missing values	1(3.4)	2 (6.5)	4 (12.9)	1 (3.3)	8 (6.6)

Note: the percentage of primary outcome missings (height, weight, MUAC, and the anthropometric indices of HAZ, WHZ, and WAZ) did not differ among the four arms (*p* = 0.534). Little’s test for height (Chi-Square = 39.952, DF = 39, Sig = 0.428), weight (Chi-Square = 74.363, DF = 73, Sig = 0.434), and MUAC (Chi-Square = 55.335, DF = 66, Sig = 0.822), suggesting a mechanism MCAR, in terms of missing data.

## Appendix E. Outcomes during the Follow-Up and by Arms

**Table A3:** Outcomes: Means and standard deviations by follow-up and arms, and differences between Fu6 and Fu1.

Outcome	Follow-Up	A1 (n = 29)	A2 (n = 31)	A3 (n = 31)	A4 (n = 30)	*p*-Value
		Mean ± SD	Mean ± SD	Mean ± SD	Mean ± SD	
**Height**	**Fu1**	86.61 ± 5.27	88.70 ± 5.84	86.92 ± 5.29	88.02 ± 6.68	0.469
	**Fu2**	89.38 ± 5.43	91.45 ± 5.82	89.90 ± 4.96	90.67 ± 6.65	0.528
	**Fu3**	91.95 ± 5.60	94.22 ± 5.50	92.40 ± 5.21	93.39 ± 6.62	0.420
	**Fu4**	94.48 ± 5.85	96.71 ± 5.35	95.18 ± 4.86	96.01 ± 6.57	0.432 *
	**Fu5**	96.83 ± 5.78	99.23 ± 5.37	97.47 ± 5.01	98.30 ± 6.82	0.408
	**Fu6**	99.29 ± 5.90	101.35 ± 5.33	99.91 ± 5.09	100.51 ± 6.46	0.548
	**Dif fu6-fu1 ^#^**	12.69 ± 2.01	12.65 ± 1.83	12.99 ± 1.72	12.49 ± 1.65	0.745
**Weight**	**Fu1**	11.83 ± 1.72	12.39 ± 1.86	12.08 ± 1.48	12.25 ± 1.91	0.572 *
	**Fu2**	12.49 ± 1.86	13.12 ± 1.83	12.88 ± 1.55	13.06 ± 1.94	0.615 *
	**Fu3**	13.20 ± 1.78	13.82 ± 1.84	13.56 ± 1.70	13.66 ± 2.03	0.644 *
	**Fu4**	13.85 ± 1.91	14.45 ± 1.84	14.25 ± 1.65	14.37 ± 2.01	0.580 *
	**Fu5**	14.44 ± 2.13	14.87 ± 1.82	14.75 ± 1.67	14.79 ± 2.20	0.798 *
	**Fu6**	14.67 ± 2.27	15.32 ± 1.92	15.13 ± 1.77	15.36 ± 2.29	0.618 *
	**Dif fu6-fu1 ^#^**	2.83 ± 0.95	2.93 ± 0.89	3.05 ± 0.76	3.11 ± 0.73	0.579
**HAZ**	**Fu1**	−1.33 ± 1.18	−1.29 ± 1.21	−1.33 ± 1.16	**−1.42 ± 1.19**	0.981
	**Fu2**	−1.31 ± 1.19	−1.23 ± 1.24	−1.25 ± 1.04	−1.38 ± 1.19	0.962
	**Fu3**	−1.28 ± 1.21	−1.13 ± 1.06	−1.24 ± 1.08	−1.30 ± 1.15	0.941
	**Fu4**	−1.24 ± 1.22	−1.08 ± 1.00	−1.12 ± 0.99	−1.21 ± 1.12	0.937
	**Fu5**	−1.19 ± 1.14	−0.99 ± 1.02	−1.11 ± 1.00	−1.19 ± 1.15	0.874
	**Fu6**	−1.12 ± 1.14	**−0.99 ± 0.98**	−1.05 ± 1.02	−1.15 ± 1.07	0.924
	**Dif fu6-fu1 ^#^**	0.21 ± 0.50	0.30 ± 0.51	0.28 ± 0.38	0.26 ± 0.36	0.849
**WHZ**	**Fu1**	−0.20 ± 1.09	−0.16 ± 1.14	0.03 ± 0.75	−0.10 ± 0.89	0.793
	**Fu2**	−0.21 ± 1.04	−0.08 ± 0.96	0.10 ± 0.88	0.05 ± 1.01	0.608
	**Fu3**	−0.12 ± 0.96	−0.07 ± 1.03	**0.14 ± 0.97**	−0.04 ± 0.98	0.754
	**Fu4**	−0.11 ± 0.90	−0.07 ± 1.00	0.11 ± 0.94	0.01 ± 1.05	0.829
	**Fu5**	−0.15 ± 0.94	−0.27 ± 0.76	0.03 ± 0.86	−0.19 ± 0.91	0.579
	**Fu6**	**−0.51 ± 1.06**	−0.39 ± 0.83	−0.20 ± 0.83	−0.23 ± 0.98	0.542
	**Dif fu6-fu1 ^#^**	−0.31 ± 0.79	−0.22 ± 0.86	−0.23 ± 0.58	−0.13 ± 0.55	0.824
**WAZ**	**Fu1**	−0.86 ± 1.06	−0.81 ± 1.10	−0.69 ± 0.88	−0.84 ± 0.92	0.909
	**Fu2**	−0.87 ± 1.06	−0.75 ± 0.96	−0.62 ± 0.86	−0.73 ± 0.92	0.792
	**Fu3**	−0.80 ± 0.97	−0.69 ± 0.90	−0.60 ± 0.91	−0.76 ± 0.93	0.839
	**Fu4**	−0.79 ± 0.95	−0.68 ± 0.89	**−0.57 ± 0.85**	−0.70 ± 0.87	0.815
	**Fu5**	−0.80 ± 1.03	−0.77 ± 0.84	−0.64 ± 0.78	−0.82 ± 0.88	0.845
	**Fu6**	**−1.00 ± 1.03**	−0.84 ± 0.86	−0.76 ± 0.79	−0.84 ± 0.93	0.752
	**Dif fu6-fu1 ^#^**	−0.15 ± −0.55	−0.03 ± 0.64	−0.07 ± 0.44	0.01 ± 0.31	0.655
**MUACZ**	**Fu1**	−0.92 ± 1.11	−0.76 ± 1.10	−0.62 ± 0.65	**−0.59 ± 0.82**	0.508
	**Fu2**	−0.91 ± 0.81	−0.71 ± 0.94	−0.60 ± 0.61	−0.71 ± 0.83	0.518
	**Fu3**	**−0.95 ± 0.85**	−0.78 ± 0.92	−0.81 ± 0.74	−0.66 ± 0.73	0.591
	**Fu4**	−0.85 ± 0.81	−0.72 ± 1.02	−0.67 ± 0.59	−0.64 ± 0.67	0.733
	**Fu5**	−0.87 ± 0.92	−0.80 ± 0.88	−0.64 ± 0.72	−0.71 ± 0.78	0.736
	**Fu6**	−0.93 ± 0.90	−0.91 ± 0.75	−0.88 ± 0.68	−0.85 ± 0.68	0.977
	**Dif fu6-fu1 ^#^**	−0.01 ±0.75	−0.15 ± 0.89	−0.26 ± 0.67	−0.26 ± 0.52	0.496

From a descriptive point of view, children in A2 were, on average, slightly taller (and older) at entry and persisted with higher mean heights throughout the study. Children in A1 were, on overage, slightly thinner (and younger) at entry and persisted with lower mean weights throughout the study compared to the remaining groups. Comparing the mean HAZ, WHZ, and WAZ from Fu1 to Fu6, no differences were detected among arms. The mean HAZ was slightly higher in A1 compared to the remaining arms. However, after two years, slight improvements in mean HAZ were registered mainly in A2, followed by A3, A4, and A1, although mean values remained negative and far from zero in all six moments (ranging from −1.42 ± 1.19 and −0.99 ± 0.98). mean values of WHZ during follow-up were higher compared with HAZ (ranging from −0.51±1.06 and 0.14 ± 0.97), with slight improvements until Fu4, followed by a decreased close to those values observed at the baseline. Mean values of WAZ ranged from −1.00 ± 1.03 to −0.57 ± 0.85 and remained negative and without significant differences throughout the study. Mean values of MUACZ remained negative across follow-up period, ranging from −0.59 ± 0.82 to −0.95 ± 0.85. * Kruskal–Wallis; # difference between initial follow-up (Fu1) and final follow-up (Fu6).

**Table A4:** Absolute (n) and relative (%) frequencies of stunting, wasting and underweight in children during the follow-up and by arms, and differences between Fu6 and Fu1.

Variables		Follow-Up	A1	A2	A3	A4	*p*-Value
			(n = 29)	(n = 31)	(n = 31)	(n = 30)	
			n (%)	n (%)	n (%)	n (%)	
**Stunting**	**Moderate-to-severe** (HAZ < −2)	**Fu1**	7 (24.1)	11 (35.5)	8 (25.8)	11 (**36.7**)	0.630
		**Fu2**	7 (24.1)	10 (32.3)	7 (22.6)	10 (33.3)	0.727
		**Fu3**	8 (27.6)	6 (**19.4**)	9 (29.0)	11 (**36.7**)	0.526
		**Fu4**	7 (24.1)	7 (22.6)	7 (22.6)	10 (33.3)	0.741
		**Fu5**	7 (24.1)	6 (19.4)	8 (25.8)	9 (30.0)	0.807
		**Fu6**	6 (20.7)	6 (19.4)	7 (22.6)	8 (26.7)	0.914
		***p*-value (Fu6 vs. Fu1) ^§^**	1.000	0.063	1.000	0.375	
		**Dif (%) Fu6—Fu1 ^#^**	−3.4	−16.1	−3.2	−10.0	
	**Mild-to-severe** (HAZ < −1)	**Fu1**	21 (72.4)	21 (67.7)	21 (67.7)	20 (66.7)	0.966
		**Fu2**	15 (51.7)	20 (64.5)	21 (67.7)	20 (66.7)	0.570
		**Fu3**	15 (51.7)	17 (54.8)	20 (64.5)	18 (60.0)	0.762
		**Fu4**	15 (51.7)	16 (51.6)	19 (61.3)	16 (53.3)	0.865
		**Fu5**	15 (51.7)	17 (54.8)	17 (54.8)	14 (46.7)	0.918
		**Fu6**	13 (44.8)	15 (48.4)	17 (54.8)	14 (46.7)	0.891
		***p*-value (Fu6 vs. Fu1) ^§^**	0.008 *	0.031 *	0.125	0.031 *	
		**Dif (%) Fu6—Fu1 ^#^**	**−27.6**	**−19.3**	−12.9	**−20.0**	
**Wasting**	**Moderate-to-severe** (WHZ < −2)	**Fu1**	1 (3.4)	2 (6.5)	0 (0.0)	1 (3.3)	0.652 ^¶^
		**Fu2**	1 (3.4)	0 (0.0)	0 (0.0)	0 (0.0)	0.240 ^¶^
		**Fu3**	1 (3.4)	1 (3.2)	1 (3.2)	2 (6.7)	0.874 ^¶^
		**Fu4**	1 (3.4)	1 (3.2)	1 (3.2)	1 (3.3)	1.000 ^¶^
		**Fu5**	2 (6.9)	2 (6.5)	0 (0.0)	0 (0.0)	0.257
		**Fu6**	2 (6.9)	0 (0.0)	0 (0.0)	2 (6.7)	0.213
		***p*-value (Fu6 vs. Fu1) ^§^**	1.000			1.000	
		**Dif (%) Fu6—Fu1 ^#^**	3.5	−6.5	0.0	3.4	
	**Mild-to-severe** (WHZ < −1)	**Fu1**	8 (27.6)	7 (22.6)	1 (3.2)	4 (13.3)	**0.038 ^¶^**
		**Fu2**	7 (24.1)	3 (9.7)	3 (9.7)	5 (16.7)	0.359 ^¶^
		**Fu3**	7 (24.1)	4 (12.9)	2 (6.5)	4 (13.3)	0.282 ^¶^
		**Fu4**	5 (17.2)	4 (12.9)	3 (9.7)	6 (20.0)	0.668 ^¶^
		**Fu5**	6 (20.7)	4 (12.9)	2 (6.5)	8 (26.7)	0.157 ^¶^
		**Fu6**	11 (37.9)	7 (22.6)	3 (9.7)	6 (20.0)	0.079 ^¶^
		***p*-value (Fu6 vs. Fu1) ^§^**	0.250	1.000	0.500	0.500	
		**Dif (%) Fu6—Fu1 ^#^**	10.3	0.0	6.5	6.7	
**Underweight**	**Moderate-to-severe** (WAZ < −2)	**Fu1**	4 (13.8)	4 (12.9)	1 (3.2)	3 (10.0)	0.474
		**Fu2**	4 (13.8)	3 (9.7)	2 (6.5)	3 (10.0)	0.786
		**Fu3**	4 (13.8)	1 (3.2)	2 (6.5)	3 (10.0)	0.415
		**Fu4**	2 (6.9)	3 (9.7)	0 (0.0)	3 (10.0)	0.315
		**Fu5**	3 (10.3)	2 (6.5)	0 (0.0)	2 (6.7)	0.344
		**Fu6**	5 (17.2)	2 (6.5)	1 (3.2)	3 (10.0)	0.256
		***p*-value (Fu6 vs. Fu1) ^§^**	1.000	1.000	1.000	1.000	
		**Dif (%) Fu6—Fu1 ^#^**	3.4	−6.4	0.0	0.0	
	**Mild-to-severe** (WAZ < −1)	**Fu1**	10 (34.5)	13 (41.9)	11 (35.5)	14 (46.7)	0.757
		**Fu2**	10 (34.5)	12 (38.7)	10 (32.3)	10 (33.3)	0.962
		**Fu3**	11 (37.9)	12 (38.7)	11 (35.5)	11 (36.7)	1.000
		**Fu4**	11 (37.9)	10 (32.3)	10 (32.3)	11 (36.7)	0.951
		**Fu5**	11 (37.9)	15 (48.4)	11 (35.5)	13 (43.3)	0.736
		**Fu6**	14 (48.3)	14 (45.2)	12 (38.7)	13 (43.3)	0.903
		***p*-value (Fu6 vs. Fu1) ^§^**	0.125	1.000	1.000	1.000	
		**Dif (%) Fu6—Fu1 ^#^**	13.8	3.3	3.2	−3.4	

^§^ McNemar’s test; ^¶^ Fisher’s exact test; ^#^ Difference in percentage between initial (Fu1) and final follow-up (Fu6); * *p* < 0.05.

**Table A5:** Absolute (n) and relative (%) frequencies of stunting during the follow-up and by gender, and comparison between Fu1 and Fu6.

Gender	Variable	Follow-Up	A1 (n = 29)	A2 (n = 31)	A3 (n = 31)	A4 (n = 30)	*p*-Value
			n (%)	n (%)	n (%)	n (%)	
**Female** (N = 61)	**Moderate-to-severe stunting** (HAZ < −2)	**Fu1**	3 (23.1)	6 (46.2)	4 (22.2)	5 (29.4)	0.540
		**Fu2**	3 (23.1)	6 (46.2)	3 (16.7)	3 (17.6)	0.266
		**Fu3**	4 (30.8)	3 (23.1)	4 (22.2)	4 (23.5)	0.954
		**Fu4**	3 (23.1)	4 (30.8)	2 (11.1)	3 (17.6)	0.597
		**Fu5**	3 (23.1)	3 (23.1)	3 (16.7)	3 (17.6)	0.947
		**Fu6**	2 (15.4)	3 (23.1)	3 (16.7)	2 (11.8)	0.934
		***p*-value *** **(Fu1 vs. Fu6)**	1.000	0.250	1.000	0.250	
	**Mild-to-severe stunting** (HAZ < −1)	**Fu1**	10 (76.9)	9 (69.2)	12 (66.7)	11 (64.7)	0.929
		**Fu2**	8 (61.5)	9 (69.2)	11 (61.1)	11 (64.7)	0.983
		**Fu3**	9 (69.2)	8 (61.5)	11 (61.1)	9 (52.9)	0.847
		**Fu4**	9 (69.2)	9 (69.2)	11 (61.1)	7 (41.2)	0.357
		**Fu5**	9 (69.2)	9 (69.2)	10 (55.6)	7 (41.2)	0.359
		**Fu6**	7 (53.8)	8 (61.5)	10 (55.6)	6 (35.3)	0.532
		***p*-value *** **(Fu1 vs. Fu6)**	0.250	1.000	0.500	0.063	
**Male** (N = 60)	**Moderate-to-severe stunting** (HAZ < −2)	**Fu1**	4 (25.0)	5 (27.8)	4 (30.8)	6 (46.2)	0.283
		**Fu2**	4 (25.0)	4 (22.2)	4 (30.8)	7 (53.8)	0.160
		**Fu3**	4 (25.0)	3 (16.7)	5 (38.5)	7 (53.8)	0.160
		**Fu4**	4 (25.0)	3 (16.7)	5 (38.5)	7 (53.8)	0.160
		**Fu5**	4 (25.0)	3 (16.7)	5 (38.5)	6 (46.2)	0.299
		**Fu6**	4 (25.0)	3 (16.7)	4 (30.8)	6 (46.2)	0.359
		***p*-value *** **(Fu1 vs. Fu6)**	1.000	0.500	1.000	1.000	
	**Mild-to-severe stunting** (HAZ < −1)	**Fu1**	11 (68.8)	12 (66.7)	9 (69.2)	9 (69-2)	1.000
		**Fu2**	7 (43.8)	11 (61.1)	10 (76.9)	9 (69.2)	0.315
		**Fu3**	6 (37.5)	9 (50.0)	9 (69.2)	9 (69.2)	0.261
		**Fu4**	6 (37.5)	7 (38.9)	8 (61.5)	9 (69.2)	0.219
		**Fu5**	6 (37.5)	8 (44.4)	7 (53.8)	7 (53.8)	0.757
		**Fu6**	6 (37.5)	7 (38.9)	7 (53.8)	8 (61.5)	0.515
		***p*-value *** **(Fu1 vs. Fu6)**	0.063	0.063	0.500	1.000	

* McNemar’s test.

## Appendix F. Description of Parasitic Infection Rates during the Follow-Up Period in Children Allocated to A3 and A4

Between Fu1 and Fu2 parasitic infections decreased from 42.3% (11/26) to 36.4% (8/22) in A3, and from 58.3% (14/24) to 33.3% (8/24) in A4. After, in Fu3, an increase of positive results was observed, achieving 56.0% (14/25) in A3 and 53.8% (14/26) in A4. From that moment on, positive cases slightly declined until the final follow-up (Fu6), reaching percentages of 39.1% (9/23) and 33.3% (8/24) in A3 and A4, respectively.

Similar variation in *G. lamblia* infection was observed in A3 across follow-ups—Fu1 (34.6%), Fu2 (18.2%), Fu3 (44.0%), Fu4 (29.2%), Fu5 (27.3%), and Fu6 (30.4%), and in A4—Fu1 (37.5%), Fu2 (12.5%), Fu3 (26.9%), Fu4 (25.0%), Fu5 (24.0%), and Fu6 (16.7%). The reduction of *Giardia* infection was not significant in neither A3 (*p* = 0.727) nor A4 (*p* = 0.07).

Regarding *A. lumbricoides* infection, lower infection rates were found in A3—Fu1 (3.8%), Fu2 (4.5%), Fu3 (4.0%), Fu4 (4.2%), Fu5 (13.6%), and Fu6 (8.7%)—compared with A4—Fu1 (4.2%), Fu2 (12.5%), Fu3 (23.1%), Fu4 (16.7%), Fu5 (20.0%), and Fu6 (8.3%), although without significant differences.

## References

[1] Black R.E., Victora C.G., Walker S.P., Bhutta Z.A., Christian P., de Onis M., Ezzati M., Grantham-McGregor S., Katz J., Martorell R. (2013). Maternal and child undernutrition and overweight in low-income and middle-income countries. Lancet.

[2] Black R.E., Allen L.H., Bhutta Z.A., Caulfield L.E., de Onis M., Ezzati M., Mathers C., Rivera J. (2008). Maternal and child undernutrition: Global and regional exposures and health consequences. Lancet.

[3] United Nations Children’s Fund (UNICEF), World Health Organization (WHO), The World Bank (2019). Levels and Trends in Child Malnutrition: Key Findings of the 2019 Edition of the Joint Child.

[4] Victora C.G., de Onis M., Hallal P.C., Blossner M., Shrimpton R. (2010). Worldwide timing of growth faltering: Revisiting implications for interventions. Pediatrics.

[5] Local Burden of Disease Child Growth Failure Collaborators (2020). Mapping child growth failure across low- and middle-income countries. Nature.

[6] Alum A., Rubino J.R., Ijaz M.K. (2010). The global war against intestinal parasites—Should we use a holistic approach?. Int. J. Infect. Dis..

[7] World Health Organization (WHO) (2017). Guideline: Preventive Chemotherapy to Control Soil-Transmitted Helminth Infections in At-Risk Population Groups.

[8] Rogawski E.T., Bartelt L.A., Platts-Mills J.A., Seidman J.C., Samie A., Havt A., Babji S., Trigoso D.R., Qureshi S., Shakoor S. (2017). Determinants and Impact of Giardia Infection in the First 2 Years of Life in the MAL-ED Birth Cohort. J. Pediatric Infect Dis Soc.

[9] Squire S.A., Ryan U. (2017). Cryptosporidium and Giardia in Africa: Current and future challenges. Parasit Vectors.

[10] Agadjanian V., Prata N. (2003). Civil war and child health: Regional and ethnic dimensions of child immunization and malnutrition in Angola. Soc. Sci. Med..

[11] República de Angola and Ministério da Saúde (MINSA) (2012). Plano Nacional de Desenvolvimento Sanitário 2012–2025. Mais e Melhor Saúde.

[12] Instituto Nacional de Estatística (INE) (2017). Inquérito de Indicadores Múltiplos e de Saúde em Angola 2015–2016.

[13] Rosario E.V., Costa D., Timoteo L., Rodrigues A.A., Varanda J., Nery S.V., Brito M. (2016). Main causes of death in Dande, Angola: Results from Verbal Autopsies of deaths occurring during 2009–2012. BMC Public Health.

[14] Sousa-Figueiredo J.C., Gamboa D., Pedro J.M., Fancony C., Langa A.J., Magalhaes R.J., Stothard J.R., Nery S.V. (2012). Epidemiology of malaria, schistosomiasis, geohelminths, anemia and malnutrition in the context of a demographic surveillance system in northern Angola. PLoS ONE.

[15] Gasparinho C., Mirante M.C., Centeno-Lima S., Istrate C., Mayer A.C., Tavira L., Nery S.V., Brito M. (2016). Etiology of Diarrhea in Children Younger Than 5 Years Attending the Bengo General Hospital in Angola. Pediatr. Infect. Dis. J..

[16] República de Angola and Ministério da Saúde (MINSA), Direcção Nacional de Saúde Pública, Departamento de Controlo de Doenças, Secção Nacional de Controlo das Doenças Tropicais Negligenciadas (2017). Plano Estratégico Nacional de Doenças Tropicais Negligenciadas 2017–2021.

[17] Croke K., Hicks J.H., Hsu E., Kremer M., Miguel E. (2017). Should the WHO withdraw support for mass deworming?. PLoS Negl. Trop. Dis..

[18] Taylor-Robinson D.C., Maayan N., Soares-Weiser K., Donegan S., Garner P. (2012). Deworming drugs for soil-transmitted intestinal worms in children: Effects on nutritional indicators, haemoglobin, and school performance. Cochrane Database Syst. Rev..

[19] Welch V.A., Ghogomu E., Hossain A., Awasthi S., Bhutta Z.A., Cumberbatch C., Fletcher R., McGowan J., Krishnaratne S., Kristjansson E. (2017). Mass deworming to improve developmental health and wellbeing of children in low-income and middle-income countries: A systematic review and network meta-analysis. Lancet Glob. Health.

[20] Taylor-Robinson D.C., Maayan N., Donegan S., Chaplin M., Garner P. (2019). Public health deworming programmes for soil-transmitted helminths in children living in endemic areas. Cochrane Database Syst. Rev..

[21] Awasthi S., Peto R., Read S., Richards S.M., Pande V., Bundy D., DEVTA (Deworming and Enhanced Vitamin A) team (2013). Population deworming every 6 months with albendazole in 1 million pre-school children in North India: DEVTA, a cluster-randomised trial. Lancet.

[22] Lo N.C., Snyder J., Addiss D.G., Heft-Neal S., Andrews J.R., Bendavid E. (2018). Deworming in pre-school age children: A global empirical analysis of health outcomes. PLoS Negl. Trop. Dis..

[23] Andrews J.R., Bogoch I.I., Utzinger J. (2017). The benefits of mass deworming on health outcomes: New evidence synthesis, the debate persists. Lancet Glob. Health.

[24] Rosario E.V.N., Costa D., Francisco D., Brito M. (2017). HDSS Profile: The Dande Health and Demographic Surveillance System (Dande HDSS, Angola). Int. J. Epidemiol..

[25] World Health Organization (WHO) (2006). WHO Child Growth Standards: Length/Height for Age, Weight-for-Age, Weight-for-Length, Weight-for-Height and Body Mass Index-for-Age, Methods and Development.

[26] World Health Organization (WHO) (2010). Nutrition Landscape Information System (NLIS) Country Profile Indicators: Interpretation Guide.

[27] World Health Organization (WHO), United Nations Children’s Fund (UNICEF) (2009). WHO Child Growth Standards and the Identification of Severe Acute Malnutrition in Infants and Children.

[28] World Health Organization (WHO) (1994). Bench Aids for the Diagnosis of Intestinal Parasites.

[29] Crompton D.W.T. (2006). Preventive Chemotherapy in Human Helminthiasis: Coordinated Use of Anthelminthic Drugs in Control Interventions: A Manual for Health Professionals and Programme Managers.

[30] De Onis M., Dewey K.G., Borghi E., Onyango A.W., Blossner M., Daelmans B., Piwoz E., Branca F. (2013). The World Health Organization’s global target for reducing childhood stunting by 2025: Rationale and proposed actions. Matern. Child Nutr..

[31] Moher D., Hopewell S., Schulz K.F., Montori V., Gøtzsche P.C., Devereaux P.J., Elbourne D., Egger M., Altman D.G. (2010). CONSORT 2010 Explanation and Elaboration: Updated guidelines for reporting parallel group randomised trials. BMJ.

[32] Chakraborty H., Gu H. (2009). A Mixed Model Approach for Intent-to-Treat Analysis in Longitudinal Clinical Trials with Missing Values.

[33] Zhang Z. (2016). Missing data imputation: Focusing on single imputation. Ann. Transl. Med..

[34] Davis C.S. (2002). Statistical Methods for the Analysis of Repeated Measurements.

[35] Noguchi K., Gel Y.R., Brunner E., Konietschke F. (2012). nparLD: An R Software Package for the Nonparametric Analysis of Longitudinal Data in Factorial Experiments. J. Stat. Softw..

[36] Chirwa E.D., Griffiths P.L., Maleta K., Norris S.A., Cameron N. (2014). Multi-level modelling of longitudinal child growth data from the Birth-to-Twenty Cohort: A comparison of growth models. Ann. Hum. Biol..

[37] Alderman H., Headey D. (2018). The timing of growth faltering has important implications for observational analyses of the underlying determinants of nutrition outcomes. PLoS ONE.

[38] Moser W., Schindler C., Keiser J. (2017). Efficacy of recommended drugs against soil transmitted helminths: Systematic review and network meta-analysis. BMJ.

[39] Forrer A., Khieu V., Schär F., Hattendorf J., Marti H., Neumayr A., Char M.C., Hatz C., Muth S., Odermatt P. (2017). Strongyloides stercoralis is associated with significant morbidity in rural Cambodia, including stunting in children. PLoS Negl. Trop. Dis..

[40] Henriquez-Camacho C., Gotuzzo E., Echevarria J., White A.C., Terashima A., Samalvides F., Pérez-Molina J.A., Plana M.N. (2016). Ivermectin versus albendazole or thiabendazole for Strongyloides stercoralis infection. Cochrane Database Syst. Rev..

[41] Rogawski E.T., Liu J., Platts-Mills J.A., Kabir F., Lertsethtakarn P., Siguas M., Khan S.S., Praharaj I., Murei A., Nshama R. (2018). Use of quantitative molecular diagnostic methods to investigate the effect of enteropathogen infections on linear growth in children in low-resource settings: Longitudinal analysis of results from the MAL-ED cohort study. Lancet Glob. Health.

[42] Kotloff K.L., Nataro J.P., Blackwelder W.C., Nasrin D., Farag T.H., Panchalingam S., Wu Y., Sow S.O., Sur D., Breiman R.F. (2013). Burden and aetiology of diarrhoeal disease in infants and young children in developing countries (the Global Enteric Multicenter Study, GEMS): A prospective, case-control study. Lancet.

[43] Platts-Mills J.A., Babji S., Bodhidatta L., Gratz J., Haque R., Havt A., McCormick B.J., McGrath M., Olortegui M.P., Samie A. (2015). Pathogen-specific burdens of community diarrhoea in developing countries: A multisite birth cohort study (MAL-ED). Lancet Glob. Health.

[44] Oliveira Y., Oliveira L.M., Oliveira Y.L.M., Nascimento A.M.D., La Corte R., Geraldi R.M., Barbosa L., Gazzinelli-Guimarães P.H., Fujiwara R.T., Bueno L.L. (2020). Changes in the epidemiological profile of intestinal parasites after a school-based large-scale treatment for soil-transmitted helminths in a community in northeastern Brazil: Epidemiological profile after large-scale school-based treatment for STH. Acta Trop.

[45] Mascha E.J., Vetter T.R. (2018). Significance, Errors, Power, and Sample Size: The Blocking and Tackling of Statistics. Anesth. Analg..

[46] Kreidler S.M., Muller K.E., Grunwald G.K., Ringham B.M., Coker-Dukowitz Z.T., Sakhadeo U.R., Baron A.E., Glueck D.H. (2013). GLIMMPSE: Online Power Computation for Linear Models with and without a Baseline Covariate. J. Stat. Softw..

[47] Donohue M.C. (2019). Longpower: Power and Sample Size Calculations for Longitudinal Data.

[48] Dupont W.D., Plummer W.D. (1990). Power and sample size calculations. A review and computer program. Control Clin. Trials.

[49] Acharya K.P., Pathak S. (2019). Applied Research in Low-Income Countries: Why and How?. Front. Res. Metr. Anal..

[50] McGowan H. (2020). An editor’s-eye view of randomized controlled trials. World Dev..

[51] Jagtap S. (2019). Design and poverty: A review of contexts, roles of poor people, and methods. Res. Engineeing Des..

[52] Savioli L., Albonico M., Daumerie D., Lo N.C., Stothard J.R., Asaolu S., Tchuem Tchuenté L.A., Anderson R.M. (2018). Review of the 2017 WHO Guideline: Preventive chemotherapy to control soil-transmitted helminth infections in at-risk population groups. An opportunity lost in translation. PLoS Negl. Trop. Dis..

[53] Mokomane M., Kasvosve I., de Melo E., Pernica J.M., Goldfarb D.M. (2018). The global problem of childhood diarrhoeal diseases: Emerging strategies in prevention and management. Ther. Adv. Infect. Dis..

[54] Mshida H.A., Kassim N., Mpolya E., Kimanya M. (2018). Water, Sanitation, and Hygiene Practices Associated with Nutritional Status of Under-Five Children in Semi-Pastoral Communities Tanzania. Am. J. Trop. Med. Hyg..

[55] Gasparinho C., Piedade J., Mirante M.C., Mendes C., Mayer C., Vaz Nery S., Brito M., Istrate C. (2017). Characterization of rotavirus infection in children with acute gastroenteritis in Bengo province, Northwestern Angola, prior to vaccine introduction. PLoS ONE.

[56] Soares Magalhaes R.J., Langa A., Pedro J.M., Sousa-Figueiredo J.C., Clements A.C., Vaz Nery S. (2013). Role of malnutrition and parasite infections in the spatial variation in children’s anaemia risk in northern Angola. Geospat. Health.

[57] Oliveira D., Ferreira F.S., Atouguia J., Fortes F., Guerra A., Centeno-Lima S. (2015). Infection by Intestinal Parasites, Stunting and Anemia in School-Aged Children from Southern Angola. PLoS ONE.

[58] Olofin I., McDonald C.M., Ezzati M., Flaxman S., Black R.E., Fawzi W.W., Caulfield L.E., Danaei G. (2013). Associations of suboptimal growth with all-cause and cause-specific mortality in children under five years: A pooled analysis of ten prospective studies. PLoS ONE.

[59] Carrel M., Rennie S. (2008). Demographic and health surveillance: Longitudinal ethical considerations. Bull. World Health Organ.

